# Adipose tissue-derived microRNA-450a-5p induces type 2 diabetes mellitus by downregulating DUSP10

**DOI:** 10.1186/s43556-025-00247-w

**Published:** 2025-02-06

**Authors:** Jiaojiao Zhu, Yanting Hou, Wei Yu, Jingzhou Wang, Xiaolong Chu, Xueting Zhang, Huai Pang, Dingling Ma, Yihan Tang, Menghuan Li, Chenggang Yuan, Jianxin Xie, Cuizhe Wang, Jun Zhang

**Affiliations:** 1 Medical College of Shihezi University, Bei-Er-Lu, Shihezi, Xinjiang, 832000 China; 2 School of Pharmacy, Xinjiang Shihezi University, Xinjiang, 832002 China

**Keywords:** Obesity, microRNA-450a-5p, DUSP10, T2DM

## Abstract

Type 2 diabetes mellitus (T2DM) has rapidly increased worldwide, emerging as the fifth leading cause of death. The treatment of T2DM is challenging due to the side effects of oral hypoglycemic drugs and the limited efficacy of long-term insulin therapy, which can lead to insulin resistance (IR). Consequently, there is significant in discovering new drugs that have minimal side effects and a pronounced hypoglycemic effect. In obesity, microRNA levels have been implicated in glucose metabolism disorders and T2DM, although many aspects remain unresolved. Here, we confirmed that visceral adipose tissue and serum microRNA-450a-5p content increased under obesity and T2DM, and it was significantly positively associated with fasting blood glucose, triglycerides, cholesterol, low-density lipoproteins-cholesterol levels of the subjects. In high-fat diet (HFD)-induced obese mice, microRNA-450a-5p expression was increased in the serum, liver, and white adipose tissue. Moreover, the adipose *Dicer*-knockout mouse model was constructed to identify adipose tissue as the main source of microRNA-450a-5p. microRNA-450a-5p could inactivate the insulin signal pathway by targeting the inhibited Dual Specificity Phosphatase 10 (DUSP10) and inducing IR and glucose metabolism disorders in vitro cultured hepatocytes and adipocytes. Additionally, microRNA-450a-5p was found to regulate DUSP10 expression and insulin signaling activity, influencing glucose tolerance and insulin sensitivity across various models, including normal diet, HFD-induced obese, adipose tissue-specific microRNA-450a-5p-knockout, and db/db mice. Furthermore, gallic acid might play a potential role in inhibiting glucose levels by decreasing microRNA-450a-5p expression. Thus, microRNA-450a-5p emerges as an attractive therapeutic target for addressing obesity, IR, and T2DM.

**Supplementary Information:**

The online version contains supplementary material available at 10.1186/s43556-025-00247-w.

## Introduction

Type 2 diabetes mellitus (T2DM) is an endocrine disease characterized by hyperglycemia and insulin resistance (IR) to target tissues, which has grown rapidly globally and has become the fifth leading cause of death worldwide [1–3]. The treatment of T2DM presents significant challenges due to the side effects associated with oral hypoglycemic drugs and the limited efficacy of long-term insulin therapy, which can result in insulin IR [4]. Consequently, many current studies aim to elucidate the molecular mechanisms of T2DM and develop new drugs with minimal side effects and significant hypoglycemic effects, thereby advancing the prevention and treatment of T2DM, but there are still many bottlenecks that have not been solved.

In 80% of T2DM patients, obesity is an important risk factor for metabolic disorders such as T2DM and IR [5, 6]. Obesity is also a chronic metabolic disease caused by excessive lipid accumulation in adipose tissue. With leptin and adiponectin as adipose factors, adipose has been defined a new endocrine organ, and its role in the regulation of obesity-related metabolic diseases has attracted considerable attention [7, 8]. Previous studies have reported that the size of adipocytes is different in adipose tissues of different body parts, and their endocrine function also differs significantly. Furthermore, the size of adipocytes is significantly positively associated with the severity of T2DM and IR [9]. For example, in brown adipose tissue (BAT), adipocytes are small and rich in the mitochondria, having strong energy metabolism regulation and insulin sensitivity; however, visceral white adipose tissue (VAT) showed an opposite effect compared with BAT [3, 10, 11]. These studies suggested that the change in adipocyte endocrine function after obesity is a critical risk factor for the occurrence of T2DM, but the specific molecular mechanism needs to be further explored.

MicroRNAs, short non-coding RNAs that regulate gene expression post-transcriptionally, have become significant molecular candidates in various complex diseases, largely because they can simultaneously regulate hundreds of target mRNAs [12]. The maturation process of microRNAs involves the regulation by enzymes such as Drosha and Dicer. The classical mechanism through which microRNAs exert their regulatory effects on target genes is by inducing mRNA degradation or inhibiting translation. An intriguing aspect of microRNAs biology is their widespread presence in various body fluids, such as serum, plasma, saliva, and amniotic fluid [13]. Notably, microRNAs found in serum have been associated with the presence of hematologic malignancies and solid tumors [14]. Furthermore, circulating microRNAs have been linked to a range of cardiovascular disorders [15]. This widespread distribution and correlation with various diseases underscore the potential of microRNAs as biomarkers for diagnostic and prognostic purposes. Because of the biological importance of microRNA, its value in the fields of glucose metabolism, lipid metabolism, and obesity is increasing [16–19]. microRNA might be a potential adipose factor [20–23]. For example, adipose tissue-derived microRNA-155 can regulate glucose metabolism in vitro and vivo [24]. The overexpression of microRNA-143 caused by obesity inhibits insulin-stimulated AKT activation and disrupts glucose metabolism [25], and microRNA-143 is a novel regulator of T2DM [26]. Identifying key microRNAs derived from adipose tissue with different expressions and clarifying its possible molecular mechanisms for inducing glucose and lipid metabolism disorders will offer a theoretical foundation for preventing and treating obesity-induced T2DM.

Three members of the microRNA450 family, namely microRNA-450a-5p, microRNA-450a-3p, and microRNA-450b-5p, play important regulatory roles in embryonic development and tumor development [27–29]. Zhang Yan et al. reported that microRNA-450a-5p could promote rat adipocyte differentiation through targeted downregulation of WISP2 [30]. And microRNA-450a-5p eliminates methylglyoxal (MGO)-induced IR via Targeting cAMP-response element binding protein (CREB) in Human umbilical vein endothelial cells (HUVECs) [31]. These findings indicated that microRNA-450a-5p could be used as a potential target to improve IR and treat patients with diabetes related diseases. In the present study, we aimed to verify the changes in microRNA-450a-5p levels in the serum and adipose tissue of subjects in the context of obesity and T2DM, as well as in high-fat diet (HFD)-induced obese mouse models. We utilized hepatocytes and adipocytes to explore the molecular mechanisms by which microRNA-450a-5p regulates IR and T2DM. To identify the primary source of circulating microRNA-450a-5p, we constructed adipose tissue *Dicer* gene knockout mice. Additionally, we evaluated the role of adipose tissue-derived microRNA-450a-5p in IR and T2DM by creating adipose tissue-specific microRNA-450a-5p knockout mice. By employing microRNA-450a-5p inhibitors to intervene in HFD induced-obese or diabetic (db/db) mice, we assessed the feasibility of targeting microRNA-450a-5p as an intervention for IR and T2DM. Finally, we evaluated whether microRNA-450a-5p could serve as a target for the potential diabetes therapy drug, gallic acid (GA), in treating T2DM. The findings of our study establish a theoretical foundation for exploring the relationship between adipose tissue-derived microRNA and T2DM. This foundation may also inform the identification of clinical targets for drug therapy. Furthermore, our research offers valuable insights and potential targets for the treatment of obesity, IR, and T2DM.

## Results

### microRNA-450a-5p levels significantly increased in obesity and T2DM individuals

To clarify the potential role and molecular mechanism of microRNA-450a-5p in obesity and diabetes. An abdominal obese mouse model was successfully constructed after administration of 60% fat feed for 12 weeks (Fig.S1a-e). Furthermore, fasting blood glucose (FBG), Triglyceride (TG), Cholesterol (TC), Low-density lipoprotein cholesterol (LDL-C), and High-density lipoprotein cholesterol (HDL-C) levels in obese mice were significantly higher than those in normal-diet mice (Fig.S1f). Moreover, serum microRNA-450a-5p levels significantly increased in obese mice compared with that in normal-diet mice (Fig.S1g), and white adipose tissue and liver microRNA-450a-5p expression also increased in obese mice (Fig.S1h and i).

We analyzed the general data and biochemical indicators of subjects (*n* = 217) and found that the body weight, body mass index (BMI), and waist circumference (WC) were significantly higher in obesity and T2DM subjects than those of normal body weight (NC) subjects. The fasting plasm glucose (FPG) and lipid indexes of individuals were significantly different among NC, obesity, and T2DM groups (Table 1).

**Table 1:** Comparison of patient metrics and biochemical parameters among the three study groups

Testing index	NC	Ob	T2DM
case number	61	105	51
Age	43.66 ± 2.38	43.08 ± 5.37	48.84 ± 7.13
woman	30	50	29
man	31	55	22
Weight (kg)	59.36 ± 6.52	81.53 ± 9.40^***^	78.34 ± 14.9^***^
BMI	21.86 ± 1.38	31.05 ± 2.7^***^	29.21 ± 4.7^***###^
WC(cm)	78.51 ± 5.89	93.36 ± 13.4^***^	98.14 ± 12.75^***#^
FPG(mmol/L)	4.52 ± 0.68	4.50 ± 0.76	9.73 ± 3.2^***###^
TG(mmol/L)	0.89 ± 0.35	1.44 ± 1.10^**^	3.15 ± 2.09^***###^
TC(mmol/L)	4.42 ± 0.71	4.93 ± 0.95^**^	5.84 ± 1.59^***###^
LDL-C(mmol/L)	2.15 ± 0.51	2.64 ± 0.75^***^	3.04 ± 0.99^***##^
HDL-C(mmol/L)	1.60 ± 0.49	1.62 ± 0.47	1.78 ± 0.70

Values are given as the mean ± *SD*

*NC* Normal weight, *Ob* Obsity group, *T2DM* Type 2 diabetes mellitus group

^**^p < 0.01, ^***^p < 0.001 compared with NC group

^#^*p* < 0.05,  ^##^*p* < 0.01, ^###^*p* < 0.001 compared with Ob group

**Fig. 1:**
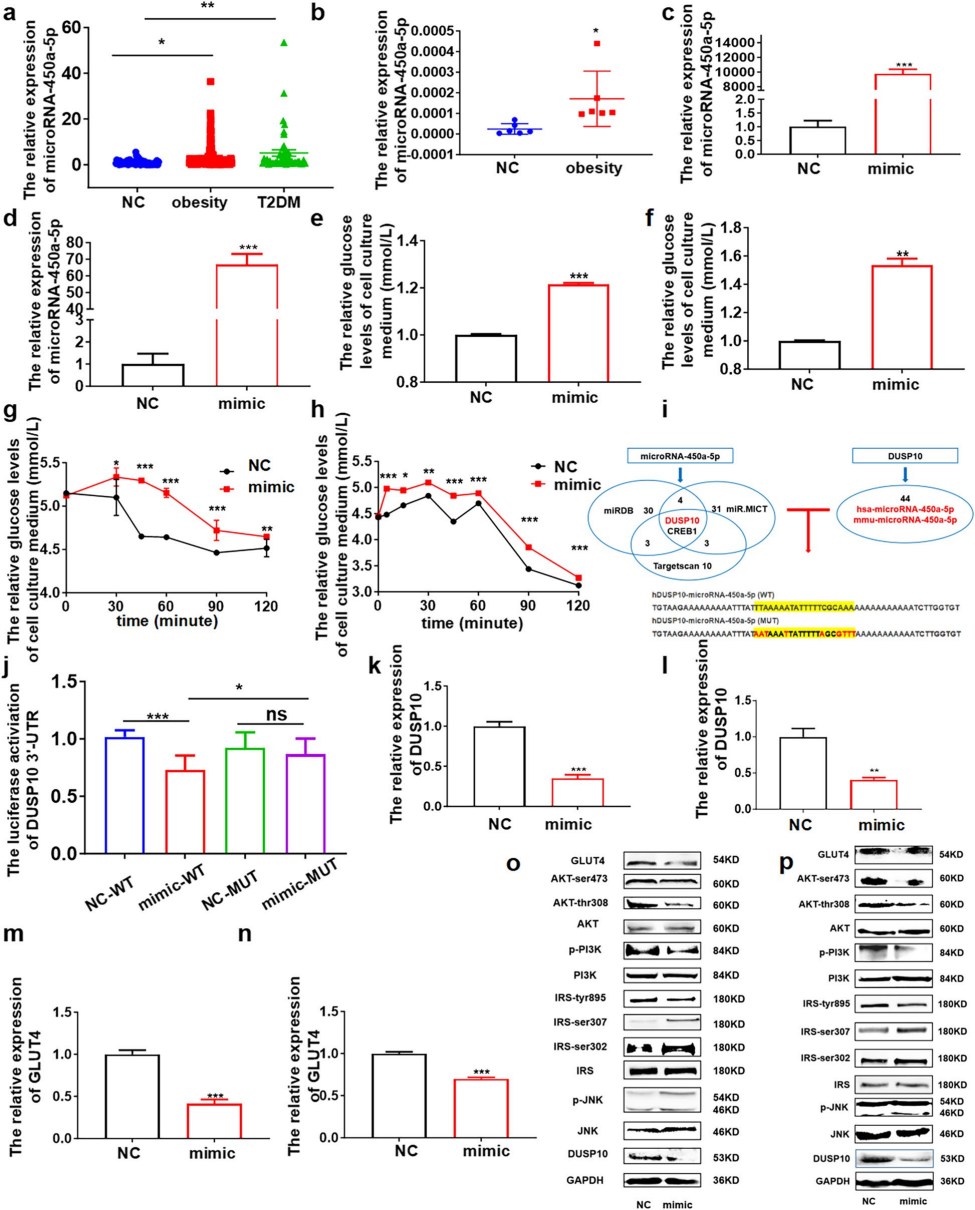
microRNA-450a-5p overexpression in hepatocytes and adipocytes impairs glucose metabolism in cells. **a** the serum microRNA-450a-5p levels of NC (*n* = 51), obesity (*n* = 105) and T2DM (*n* = 61) subjects. **b** the adipose microRNA-450a-5p levels of NC (*n* = 6), obesity (*n* = 6) subjects. **c** and **d** the expression level of microRNA-450a-5 in cells stimulated by 50 nM mimic, **c** HepG2, **d** 3T3-L1. **e** and **f** the glucose consumption, **e** HepG2, **f** 3T3-L1. **g** and **h** the insulin sensitivity, **g** HepG2, **h** 3T3-L1. **i** identified DUSP10 as the target gene of microRNA-450a-5p. **j** the luciferase activity of DUSP10 3’UTR after mimic transfected 24h. **k** and **l** the mRNA expression level of DUSP10, **k** HepG2, **l** 3T3-L1. **m** and **n** the mRNA expression level of GLUT4, **m** HepG2, **n** 3T3-L1. **o** and **p** the protein expression of DUSP10/JNK/IRS/PI3K/AKT/GLUT4, **o** HepG2, **p** 3T3-L1. ^*^p < 0.05 means a statistically significant difference

The serum microRNA-450a-5p levels in patients with obesity and T2DM were markedly increased compared with NC individuals (Fig. 1a). In addition, the content of serum microRNA-450A-5p was positively correlated with FPG, TG, TC, and LDL-C (Table 2). Moreover, microRNA-450a-5p in visceral adipose tissue of the obese group was significantly higher than that of NC (Fig. 1b). Taken together, these results suggested that microRNA-450a-5p expression was correlated with obesity and T2DM.

**Table 2:** The correlation analysis between microRNA-450a-5p and general data of patients

	r-value	*P*-value
BMI	0.113	0.098
FPG	0.247	0.0001
TG	0.259	0.0001
TC	0.204	0.01
LDL-C	0.197	0.003
HDL-C	0.067	0.174

Serum microRNA-450a-5p was measured in 217 subjects, with successful detection in 215, as detailed in Table 1. The relative expression levels of serum microRNA-450a-5p were matched with the subjects’ general data, and the results of this correlation analysis were obtained. The correlation coefficient of Spearman, *N* = 215.* p* < 0.05 means a statistically significant difference

### microRNA-450a-5p overexpression in hepatocytes and adipocytes impairs glucose metabolism

To investigate whether an increased microRNA-450a-5p expression is involved in the regulation of glucose and lipid metabolism, we performed glucose consumption, insulin sensitivity, and lipids synthesis experiments on HepG2, 3T3-L1, and L02 cells. We found that microRNA-450a-5p overexpression decreased glucose consumption and insulin sensitivity (Fig. 1c-h, Fig.S2a-d). To further explore the possible mechanism of microRNA-450a-5p in the regulation of glucose metabolism, we predicted and verified that Dual Specificity Phosphatase 10 (*DUSP10)* might be the target gene associated with glucose metabolism of microRNA-450a-5p (Fig. 1i and j). Furthermore, microRNA-450a-5p overexpression in HepG2, 3T3-L1, and L02 cells significantly decreased the expression of DUSP10 and GLUT4 at mRNA and protein levels (Fig. 1k-p, Fig.S2e-g) and the content of IRS-tyr895, p-PI3K, AKT-ser473, and AKT-thr308; and increased the content of p-JNK and IRS-ser302/307, which impaired the activity of JNK/IRS/PI3K/AKT (Fig. 1o and p, Fig.S1g). These results revealed that microRNA-450a-5p overexpression could impair glucose metabolism in vitro.

**Fig. 2:**
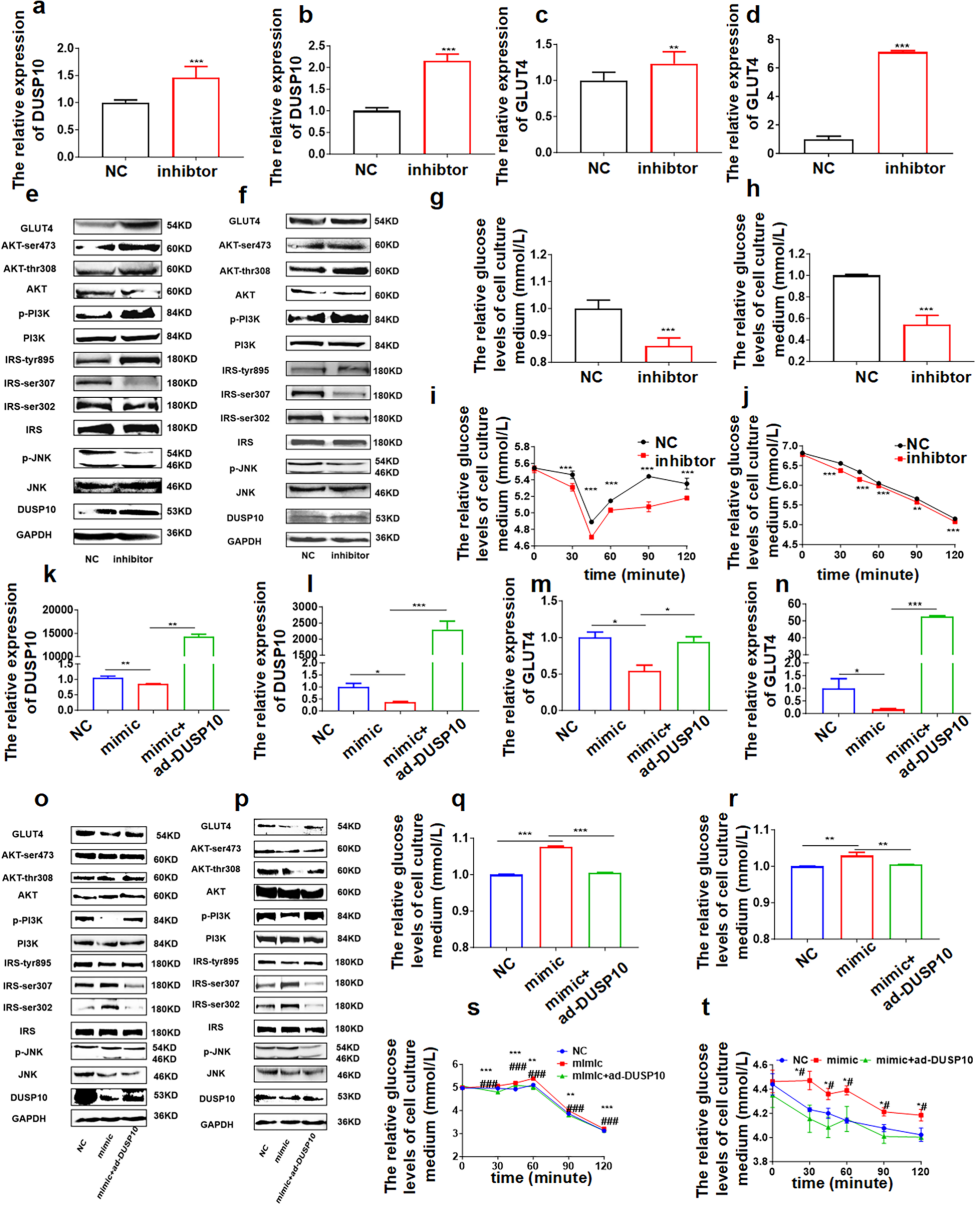
microRNA-450a-5p impairs glucose metabolism by downregulating DUSP10. After transfected HepG2 and 3T3-L1 cells with 100 nM microRNA-450a-5p inhibitor 24 h, **a** and **b** the mRNA expression level of DUSP10, **a** HepG2, **b** 3T3-L1. **c** and **d** the mRNA expression level of GLUT4, **c** HepG2, **d** 3T3-L1. **e** and **f** the protein expression of DUSP10/JNK/IRS/PI3K/AKT/GLUT4, **e** HepG2, **f** 3T3-L1. **g** and **h** the glucose consumption, **g** HepG2, **h** 3T3-L1. **i** and **j** the insulin sensitivity, **i** HepG2, **j** 3T3-L1. After transfected HepG2 and 3T3-L1 cells with 50 nM microRNA-450a-5p and DUSP10 over-expression plasmid, **k** and **l** the mRNA expression level of DUSP10, **k** HepG2, **l** 3T3-L1. **m** and **n** the mRNA expression level of GLUT4, **m** HepG2, **n** 3T3-L1. **o** and **p** the protein expression of DUSP10/JNK/IRS/PI3K/AKT/GLUT4, **o** HepG2, **p** 3T3-L1. **q** and **r** the glucose consumption, **q** HepG2, **r** 3T3-L1. **s** and **t** the insulin sensitivity, **s** HepG2, **t** 3T3-L1. Compared with NC group, ^*^p < 0.05 means a statistically significant difference, compared with mimic group, ^#^*p* < 0.05 means a statistically significant difference

**Fig. 3:**
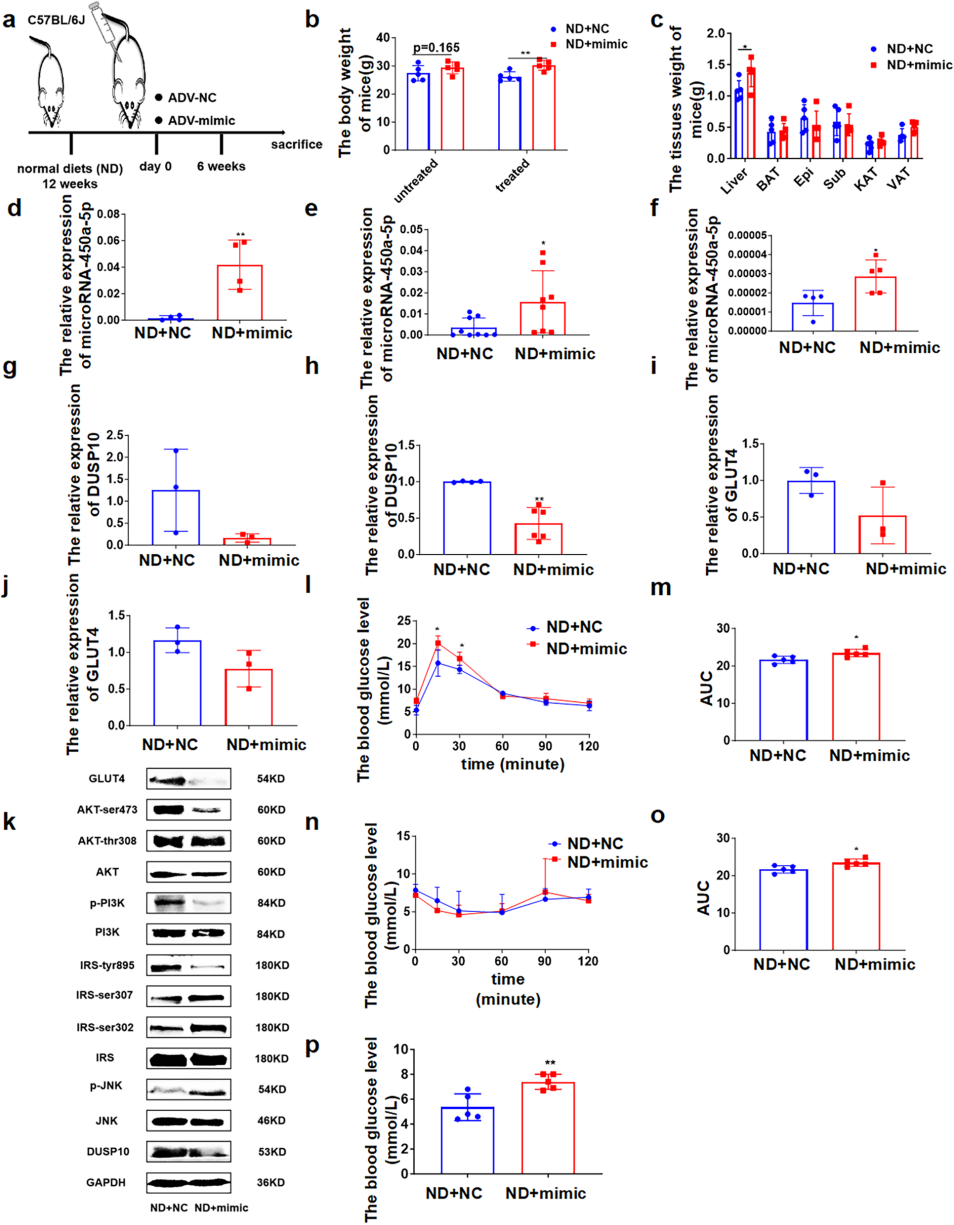
microRNA-450a-5p impairs glucose metabolism via down-regulating DUSP10 in vivo. **a** schematic diagram of intraperitoneal injection of ADV-mimic mice model construction. **b** the change of body weight. **c** the weight of brown adipose tissue (BAT), visceral adipose tissue (VAT), Perirenal adipose tissue (KAT), epididymal white adipose tissue (Epi), Subcutaneous adipose tissue (Sub) and Liver. **d** the serum microRNA-450a-5p expression level. **e** the liver microRNA-450a-5p expression level. **f** the adipose microRNA-450a-5p expression level. **g** and **h** the mRNA expression level of DUSP10 in liver (**g**) and Epi (**h**). **i** and **j** the mRNA expression level of GLUT4 in liver (**i**) and Epi (**j**). **k** the protein expression of DUSP10/JNK/IRS/PI3K/AKT/GLUT4 in liver. **l** the glucose tolerance of mice. **m** the quantitative of mice glucose tolerance. **n** the insulin sensitivity of mice. **o** the quantitative of mice insulin sensitivity. **p** the FBG levels of mice. Normal diet with ADV-NC group (ND + NC); Normal diet with ADV-mimic group (ND + mimic), ^*^p < 0.05 means a statistically significant difference

To further study the function of microRNA-450a-5p in the regulation of glucose and lipid metabolism, we inhibited microRNA-450a-5p in HepG2, 3T3-L1, and L02 cells, and the expression of DUSP10 and GLUT4 at the mRNA and protein levels were significantly increased (Fig. 2a-f, Fig.S3a-f). Moreover, the content of IRS-tyr895, p-PI3K, AKT-ser473, and AKT-thr308 was increased, whereas the content of p-JNK and IRS-ser302/307 decreased, thus increasing the activity of JNK/IRS/PI3K/AKT (Fig. 2e and f, Fig.S3f). Besides, the inhibition of microRNA-450a-5p improved glucose consumption and insulin sensitivity (Figs. 2g-j, and 3g and h). Thus, the inhibition of microRNA-450a-5p could ameliorate glucose metabolism in vitro.

### microRNA-450a-5p impairs glucose metabolism by downregulating DUSP10 in hepatocytes and adipocytes

Next, to clarify whether microRNA-450a-5p regulates glucose and lipid metabolism via DUSP10, we performed a rescue assay (overexpression of microRNA-450a-5p with DUSP10 upregulation) in HepG2, 3T3-L1, and L02 cells. The expression of DUSP10 and GLUT4 at mRNA and protein levels was significantly increased compared with microRNA-450a-5p overexpression (Fig. 2k-n, Fig.S4d-f), as well as the content of IRS-tyr895, p-PI3K, AKT-ser473, and AKT-thr308; and decreased the content of p-JNK, and IRS-ser302/307, which increased the activity of JNK/IRS/PI3K/AKT (Fig. 2o and p, Fig.S4f). These results indicated that microRNA-450a-5p impaired the activity of JNK/IRS/PI3K/AKT by downregulating DUSP10. Glucose consumption and insulin sensitivity assays showed that microRNA-450a-5p overexpression with DUSP10 upregulation could rescue a decrease in glucose consumption and insulin sensitivity induced by microRNA-450a-5p overexpression (Fig. 2q-t, Fig.S4g and h). Therefore, we speculated that an increase in microRNA-450a-5p expression in obesity models impairs glucose metabolism via DUSP10 downregulation.

### Overexpressing microRNA-450a-5p impairs glucose metabolism by downregulating DUSP10 in normal diet mice

To determine whether microRNA-450a-5p overexpression is involved in the regulation of glucose and lipid metabolism in vivo, 1 × 10^11^ adenovirus particles encoding microRNA-450a-5p-mimic were intraperitoneally injected into normal-diet mice (Fig. 3a). The body and liver weight showed a significant increase, but no change was observed in adipose tissue weight (Fig. 3b and c). An increase in microRNA-450a-5p expression was observed in the serum, liver, and adipose tissue (Fig. 3d-f). Moreover, the expression of DUSP10 and GLUT4 at mRNA and protein levels and the content of IRS-tyr895, p-PI3K, AKT-ser473, and AKT-thr308 were significantly decreased in the liver and adipose tissue, whereas the content of p-JNK and IRS-ser302/307 was increased, which decreased the activity of JNK/IRS/PI3K/AKT (Fig. 3g-k).

The intraperitoneal glucose tolerance test (IPGTT) showed that microRNA-450a-5p overexpression reduced the glucose tolerance ability (Fig. 3l and m), and the insulin sensitivity test (ITT) suggested that microRNA-450a-5p overexpression inhibited insulin sensitivity (Fig. 3n and o). Moreover, the FBG levels significantly increased with overexpression of microRNA-450a-5p (Fig. 3p). Thus, microRNA-450a-5p overexpression impaired glucose metabolism in vivo by inhibiting DUSP10 to suppress the activity of JNK/IRS/PI3K/AKT.

### Adipose tissue-derived microRNA-450a-5p impairs glucose metabolism by downregulating DUSP10 in vivo

**Fig. 4:**
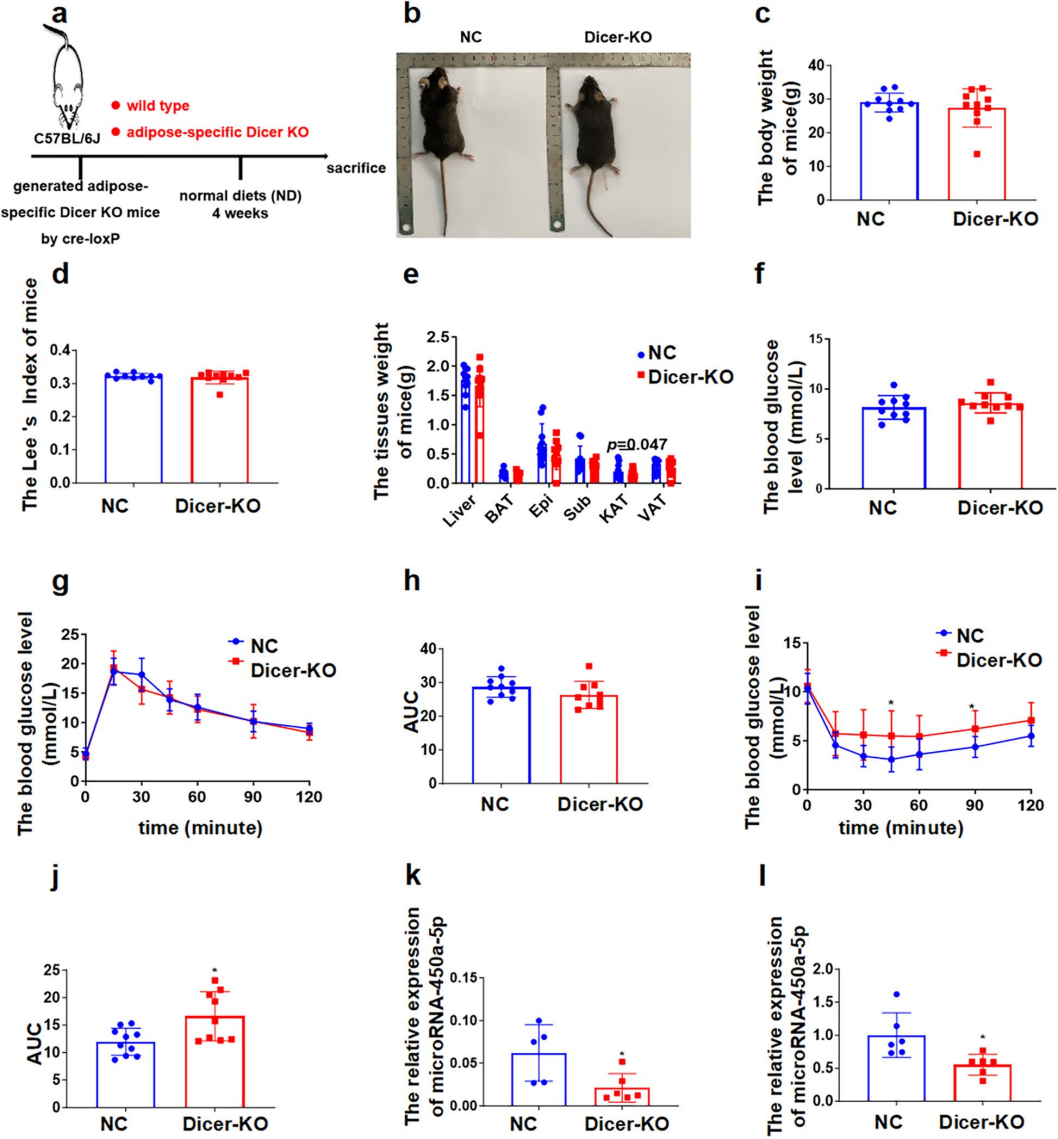
Adipose tissue is a main source of serum microRNA-450a-5p in vivo. **a** and **b** schematic diagram of adipose tissue *Dicer* knockout mice model construction. **c** the change of body weight. **d** the Lee’s index. **e** the weight of brown adipose tissue (BAT), visceral adipose tissue (VAT), Perirenal adipose tissue (KAT), epididymal white adipose tissue (Epi), Subcutaneous adipose tissue (Sub) and Liver. **f** the FBG levels of mice. **g** the glucose tolerance of mice. **h** the quantitative of mice glucose tolerance. **i** the insulin sensitivity of mice. **j** the quantitative of mice insulin sensitivity. **k** the adipose microRNA-450a-5p expression level. **l** the serum microRNA-450a-5p expression level. ^*^p < 0.05 means a statistically significant difference

Our data demonstrated that the expression of microRNA-450a-5p was upregulated in the white adipose tissue, liver, and serum of obese subjects, and many studies have suggested that adipose is the biggest endocrine organ. To identify whether microRNA-450a-5p was derived from adipose tissue, we generated adipose tissue *Dicer* conditional knockout mice using the Cre/Loxp system. qRT-PCR was performed to identify the successfully knockout of *Dicer* in adipose tissue. Their body weight, Lee’s index, liver and adipose tissue weight, blood glucose levels, glucose tolerance, and TG levels were similar to those of littermate controls (Dicer^fl/fl^) in mice (Fig. 4a-j). These data indicated that serum microRNA-450a-5p was derived from adipose tissue. Consistent with our hypothesis, the expression of microRNA-450a-5p significantly decreased in adipose tissue and serum with the deletion of Dicer in adipose tissue (Fig. 4k and l).

**Fig. 5:**
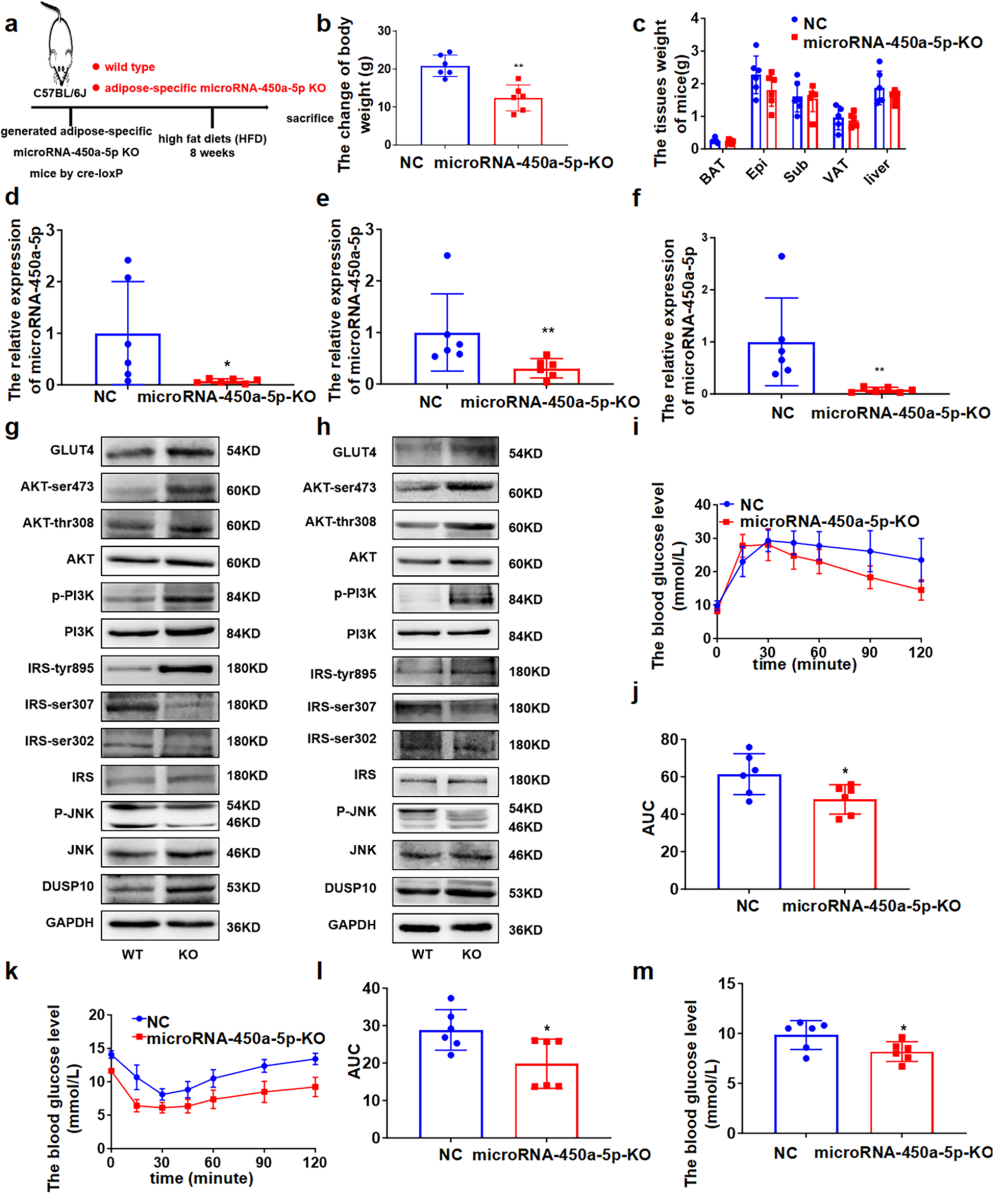
Adipose tissue derived microRNA-450a-5p impairs glucose metabolism via up-regulating DUSP10 in vivo. **a** schematic diagram of adipose tissue microRNA-450a-5p knock out mice model construction. **b** the change of body weight. **c** the weight of brown adipose tissue (BAT), visceral adipose tissue (VAT), Perirenal adipose tissue (KAT), epididymal white adipose tissue (Epi), Subcutaneous adipose tissue (Sub) and Liver. **d** the adipose microRNA-450a-5p expression level. **e** the serum microRNA-450a-5p expression level. **f** the Liver microRNA-450a-5p expression level. **g** and **h** the protein expression of DUSP10/JNK/IRS/PI3K/AKT/GLUT4 in liver (**g**) and Epi (**h**). **i** the glucose tolerance of mice. **j** the quantitative of mice glucose tolerance. **k** the insulin sensitivity of mice. **l** the quantitative of mice insulin sensitivity. **m** the FBG levels of mice. ^*^p < 0.05 means a statistically significant difference

Next, to study the function of adipose tissue-derived microRNA-450a-5p in the regulation of glucose and lipid metabolism in vivo, we generated microRNA-450a-5p conditional knockout mice using the Cre/Loxp system (Fig. 5a). The deletion of microRNA-450a-5p could inhibit the increase in body weight, liver weight, and adipose tissue weight induced by HFD to some extent (Fig. 5b and c). Expression analysis revealed an approximately 80% decrease in microRNA-450a-5p levels in microRNA-450a-5p KO versus microRNA-450a-5p^fl/fl^ adipose tissue (Fig. 5d). Moreover, it significantly reduced microRNA-450a-5p expression in the serum and liver (Fig. 5e and f). The expression of DUSP10 and GLUT4 at protein levels was significantly increased in the liver and adipose tissue compared with those of the microRNA-450a-5p^fl/fl^ group, as well as the content of IRS-tyr895, p-PI3K, AKT-ser473, and AKT-thr308 was increased; It decreased the content of p-JNK and IRS-ser302/307, which increased the activity of JNK/IRS/PI3K/AKT (Fig. 5g and h). IPGTT showed that the deletion of microRNA-450a-5p increased the glucose tolerance ability (Fig. 5i and j); ITT suggested that the deletion of microRNA-450a-5p increased insulin sensitivity (Fig. 5k and l). Moreover, FBG levels significantly decreased with the deletion of microRNA-450a-5p (Fig. 5m). Taken together, adipose tissue-derived microRNA-450a-5p impaired glucose metabolism in vivo and the deletion of microRNA-450a-5p in adipose tissue could improve glucose metabolism by increasing the expression of DUSP10 to promote the activity of JNK/IRS/PI3K/AKT.

### Inhibition of microRNA-450a-5p ameliorates glucose metabolism by upregulating DUSP10 in obese and db/db mice

**Fig. 6:**
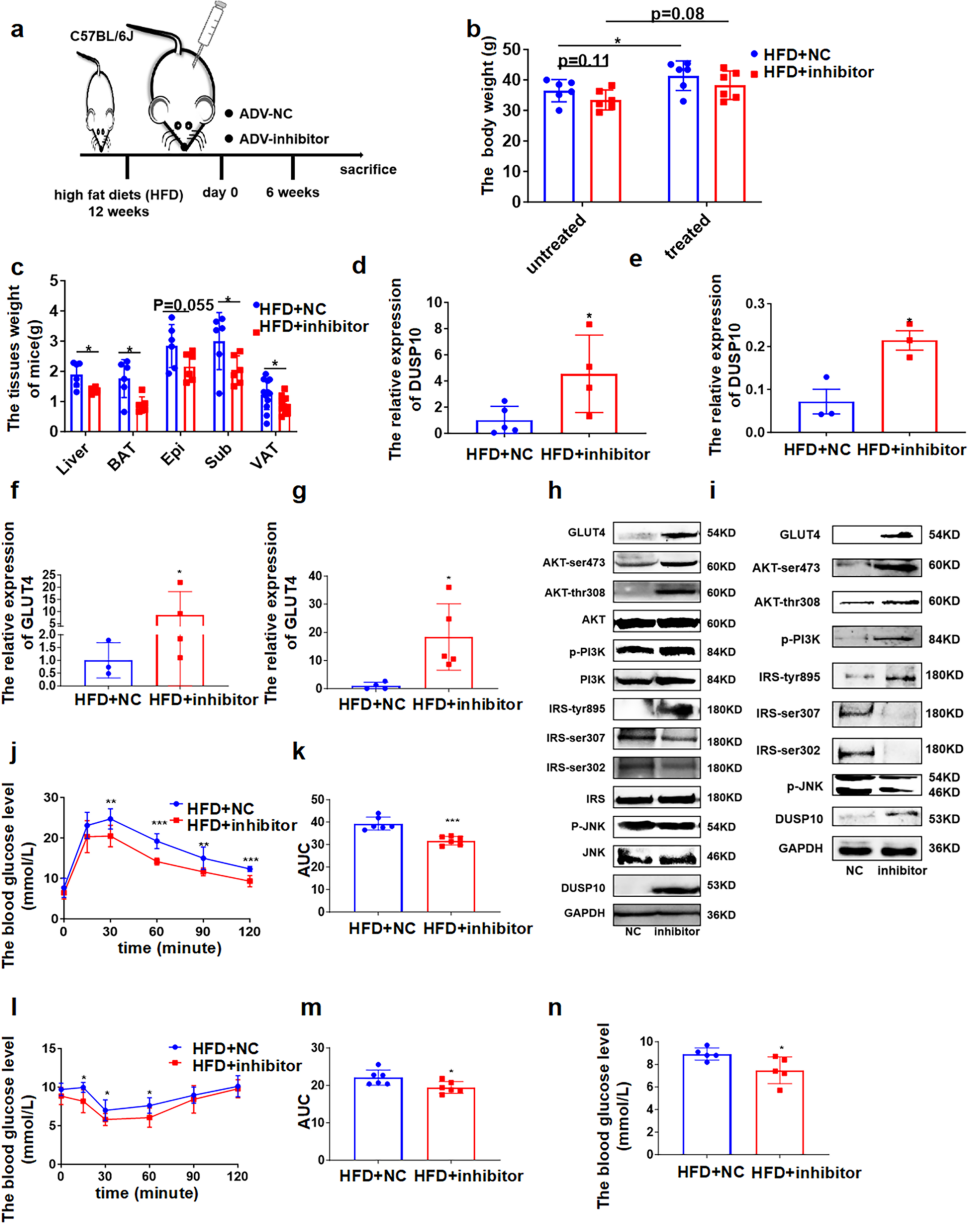
Inhibition of microRNA-450a-5p ameliorates glucose metabolism via up-regulating DUSP10 in obese mice. **a** schematic diagram of intraperitoneal injection of ADV-inhibitor in obesity mice model construction. **b** the change of body weight. **c** the weight of brown adipose tissue (BAT), visceral adipose tissue (VAT), Perirenal adipose tissue (KAT), epididymal white adipose tissue (Epi), Subcutaneous adipose tissue (Sub) and Liver. **d** and **e** the mRNA expression level of DUSP10 in liver (**d**) and Epi (**e**). **f** and **g** the mRNA expression level of GLUT4 in liver (**f**) and Epi (**g**). **h** and **i** the protein expression of DUSP10/JNK/IRS/PI3K/AKT/GLUT4 in liver (**h**) and Epi (**i**). **j** the glucose tolerance of mice. **k** the quantitative of mice glucose tolerance. **l** the insulin sensitivity of mice. **m** the quantitative of mice insulin sensitivity. **n** the FBG levels of mice. ^*^p < 0.05 means a statistically significant difference

We demonstrated that microRNA-450a-5p overexpression could impair glucose and lipids metabolism in vivo and in vitro. To identify whether the inhibition of microRNA-450a-5p can improve the decrease in glucose and lipid metabolism induced by obesity, 1 × 10^11^ adenovirus particles with microRNA-450a-5p-inhibitor were intraperitoneally injected into HFD-induced obese mice. The microRNA-450a-5p inhibitor could significantly inhibit the increase in body and liver weight induced by HFD, and the adipose tissue weight also significantly decreased. (Fig. 6a-c). The expression of DUSP10 and GLUT4 at mRNA and protein levels was significantly increased in the liver and adipose tissue compared with those of the NC group (Fig. 6d-g); the content of IRS-tyr895, p-PI3K, AKT-ser473, and AKT-thr308 was increased; the content of p-JNK and IRS-ser302/307 was decreased, which increased the activity of IRS/PI3K/AKT (Fig. 6h and i). IPGTT showed that the inhibition of microRNA-450a-5p increased the glucose tolerance ability (Fig. 6j and k), and ITT suggested that the inhibition of microRNA-450a-5p increased insulin sensitivity (Fig. 6l and m). Moreover, the FBG level significantly decreased with the inhibition of microRNA-450a-5p (Fig. 6n). The inhibition of microRNA-450a-5p ameliorated glucose metabolism in obese mice by promoting the expression of DUSP10 to increase the activity of JNK/IRS/PI3K/AKT.

**Fig. 7:**
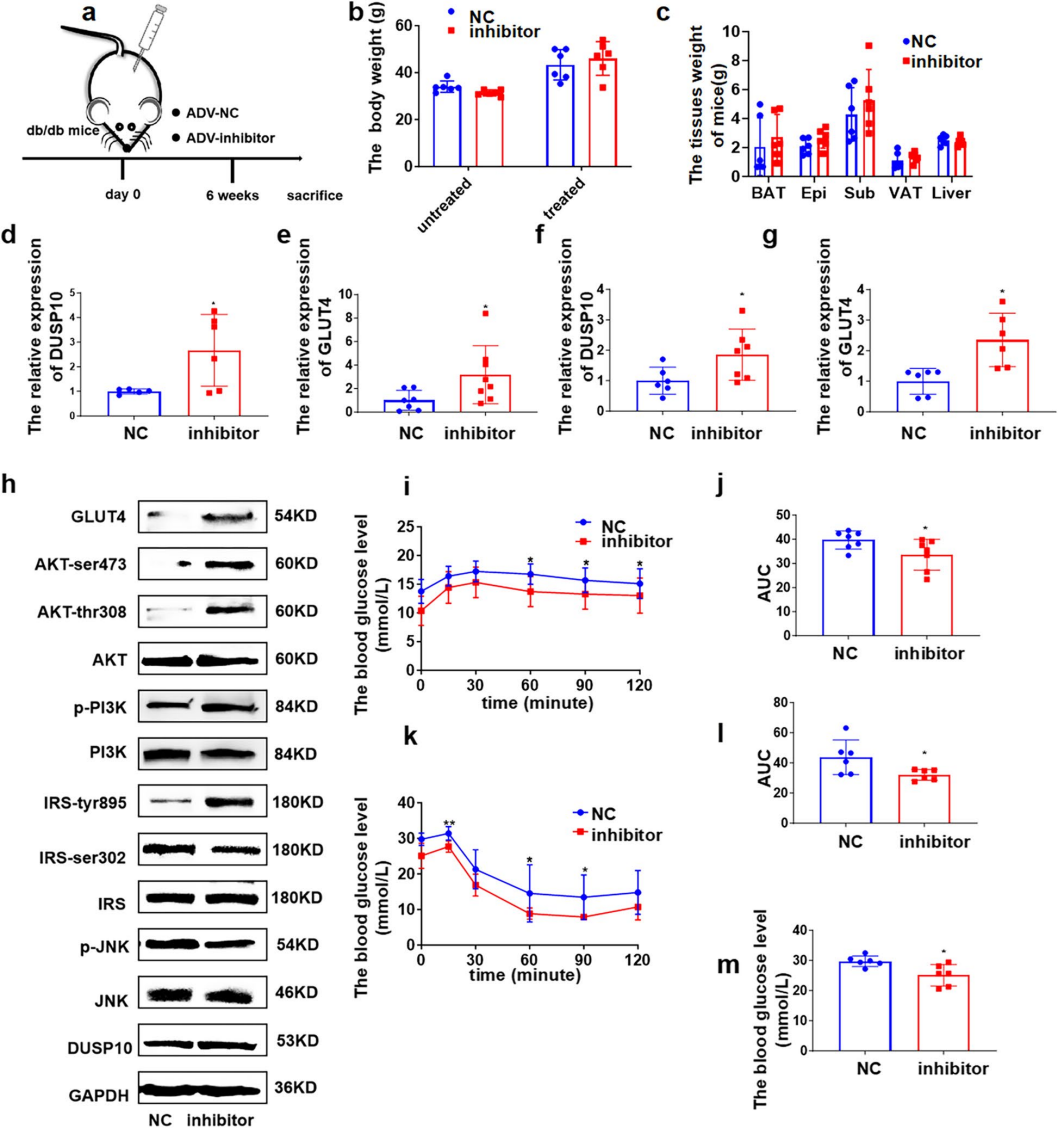
Inhibition of microRNA-450a-5p ameliorates glucose metabolism via up-regulating DUSP10 in diabetic mice. **a** schematic diagram of intraperitoneal injection of ADV-inhibitor in db/db mice model construction. **b** the change of body weight. **c** the weight of brown adipose tissue (BAT), visceral adipose tissue (VAT), Perirenal adipose tissue (KAT), epididymal white adipose tissue (Epi), Subcutaneous adipose tissue (Sub) and Liver. **d** and **e** the mRNA expression level of DUSP10 in liver (**d**) and Epi (**e**). **f** and **g** the mRNA expression level of GLUT4 in liver (**f**) and Epi (**g**). **h** the protein expression of DUSP10/JNK/IRS/PI3K/AKT/GLUT4 in liver. **i** the glucose tolerance of mice. **j** the quantitative of mice glucose tolerance. **k** the insulin sensitivity of mice. **l** the quantitative of mice insulin sensitivity. **m** the FBG levels of mice. ^*^p < 0.05 means a statistically significant difference

We next assessed the function of microRNA-450a-5p-inhibitor in the regulation of glucose homeostasis and insulin sensitivity in diabetes (db/db) mice. A total of 1 × 10^11^ adenovirus particles with microRNA-450a-5p-inhibitor were intraperitoneally injected into db/db mice, and the body, liver, and adipose tissue weight did not show a significant decrease (Fig. 7a-c). The expression of DUSP10 and GLUT4 at mRNA and protein levels was significantly increased in the liver and adipose tissue (Fig. 7d-g), as well as the content of IRS-tyr895, p-PI3K, AKT-ser473, and AKT-thr308; the content of p-JNK and IRS-ser302/307 was decreased, which increased the activity of JNK/IRS/PI3K/AKT (Fig. 7h). Moreover, it inhibited microRNA-450a-5p in db/db mice, whereas glucose tolerance and insulin sensitivity were significantly ameliorated (Fig. 7i-l). Moreover, the FBG level significantly decreased with the inhibition of microRNA-450a-5p (Fig. 7m). Collectively, these data demonstrated that the inhibition of microRNA-450a-5p ameliorated glucose metabolism in db/db mice by promoting the expression of DUSP10 to increase the activity of IRS/PI3K/AKT.

### GA downregulated microRNA-450a-5p and ameliorated glucose metabolism in obese mice

**Fig. 8:**
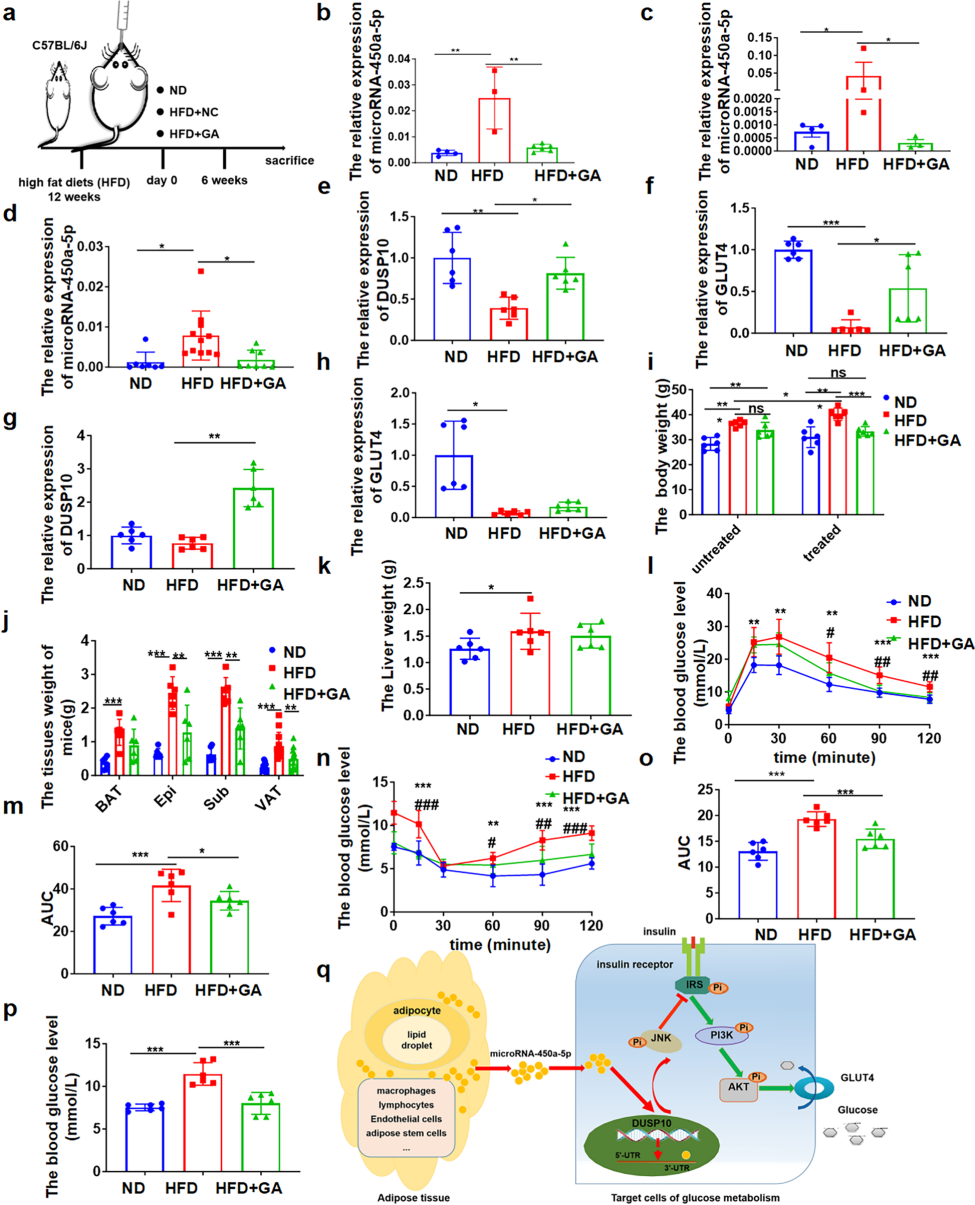
GA ameliorates glucose metabolism via down-regulating microRNA-450a-5p in obese mice. **a** schematic diagram of GA gavage in obesity mice model construction. **b** the serum microRNA-450a-5p expression level. **c** the liver microRNA-450a-5p expression level. **d** the adipose microRNA-450a-5p expression level. **e** the DUSP10 mRNA expression level in liver. **f** the GLUT4 mRNA expression level in liver. **g** the DUSP10 mRNA expression level in adipose tissue. **h** the GLUT4 mRNA expression level in adipose tissue. **i** the body weight of mice. **j** the adipose tissue weight of mice. **k** the liver weight of mice. **l** the glucose tolerance of mice. **m** the quantitative of mice glucose tolerance. **n** the insulin sensitivity of mice. **o** the quantitative of mice insulin sensitivity. **p** the FBG levels of mice. **q** a working model that illustrates the mechanism by which over-expression of microRNA-450a-5p impairs target cell glucose metabolism-targeted DUSP10. Normal diet group (ND), High fat diet group (HFD), High fat diet with GA gavage group (HFD + GA). Compared with ND group, ^*^p < 0.05 means a statistically significant difference; compared with HFD group, ^#^*p* < 0.05 means a statistically significant difference

To further explore the feasibility of microRNA-450a-5p targeting obese and type 2 diabetes mice, we used GA, known for anti-inflammatory, antitumor, and anti-oxidation biological activities, to gavage diet-induced obese mice for 6 weeks. This significantly reduced the expression of microRNA-450a-5p in the serum, liver, and adipose tissue (Fig. 8a-d). The expression of DUSP10 and GLUT4 in the liver and adipose tissue of obese mice was also upregulated after GA gavage (Fig. 8e-h). The body weight was significantly reduced compared with that of HFD-induced obese mice (Fig. 8i). The weight of white epididymal, subcutaneous, and visceral adipose tissue was significantly reduced in mice with GA gavage (Fig. 8j); however, the liver weight showed no significant change (Fig. 8k). Moreover, GA gavage could significantly ameliorate glucose tolerance and insulin sensitivity (Fig. 8l-o). Moreover, FBG levels significantly decreased with GA gavage (Fig. 8p). These findings together indicated that microRNA-450a-5p might be the target of GA in vivo, and GA could be a valuable drug for obesity and T2DM.

## Discussion

The International Diabetes Federation’s latest report predicts that the number of diabetes patients will rise to 578.4 million by 2030 and 700.2 million by 2045, significantly increasing the global health burden [32–35]. The prevalence of T2DM in Chinese adults reached 11.6%; thus, ranking the first number of new diabetes in the world, and 50.1% of the adult population were pre-diabetic [3]. Epidemiological data showed that as of 2016, China has the largest obese population globally, with 43.2 million obese men and 46.4 million obese women, representing 16.3% and 12.4% of the world’s obese population, respectively [36, 37]. A total of 80% of patients with T2DM are obese, and obesity has become an important risk factor for metabolic disorders such as T2DM and IR [5, 6]. Although several studies have identified the relationship between obesity and IR and T2DM from different perspectives, the specific molecular mechanism of its action is unclear. Due to the side effects of oral hypoglycemic drugs and a weak effect on blood glucose control, the long-term insulin treatment can lead to insulin resistance, making the treatment of T2DM challenging. Therefore, searching for new drugs with small side effects and obvious hypoglycemic effect is of great clinical significance.

In our study, analysis of serum samples and VAT of normal weight, obesity, and T2DM individuals showed that microRNA-450a-5p was significantly higher in obese and T2DM individuals than in normal-weight individuals, and microRNA-450a-5p was significantly positively correlated with FPG and blood lipids indexes of the individuals. Moreover, we verified that microRNA-450a-5p was derived from adipose tissue in adipose tissue-specific *Dicer*-knockout mice and found that glucose metabolism could be improved in adipose tissue-specific *microRNA-450a-5p*-knockout mice. The adipose tissue-derived microRNA-450a-5p was increased after obesity, probably by participating in glucose and lipid metabolic processes, thus, inducing the occurrence of T2DM. However, the specific reason for the increase in serum microRNA-450a-5p expression and its involvement in regulating glucose metabolism by regulating the key target genes is unclear. Serum TG levels increase in obesity, and TG can promote free fatty acid (FFA) synthesis via lipolysis to induce an inflammatory response in adipose tissue [38–40], whereas Sun et al. showed that IL-6 could inhibit the expression of microRNA-152. Our previous results showed that a high concentration of palmitic acid (PA) promotes IL-6 expression in adipocytes and induce inflammatory response [41, 42]. Moreover, inflammatory response induced by the increase in FFA content after obesity could impair endoplasmic function homeostasis, thus, leading to endoplasmic reticulum stress (ERS), which can be involved in the production of vesicles and exosomes to affect the release of microRNAs [43–46]. The above studies suggested that inflammatory response and ERS induced by high serum FFA content after obesity may affect the expression and release of microRNA-450a-5p. On the other hand, microRNA-450a-5p, a non-coding RNA, is regulated by transcriptional regulation, and the changes in key transcription factors after obesity may also affect the expression of microRNA-450a-5p. In addition, microRNA maturation requires the participation of multiple splicing enzymes such as Dicer enzymes, and the alterations both expression and activity of key enzymes might be involved in microRNA maturation after obesity, which may affect the expression of microRNA-450a-5p.

*DUSP10* was identified as the downstream target gene of microRNA-450a-5p using the bioinformatics database and luciferase reporter assay. DUSP10, a phosphatase enzyme, can affect glucose metabolism by regulating JNK/IRS/PI3K/AKT activity [47, 48]. However, whether microRNA-450a-5p regulates glucose metabolism in target tissues by downregulating DUSP10 has not been reported. Further review of the gene cards database revealed that the expression abundance of DUSP10 was high in the target organs of glucose metabolism, such as the liver, adipose, and skeletal muscle tissues. Moreover, microRNA-450a-5p was highly expressed in white adipose tissue and liver of obese mice, but low expressed in the kidney, spleen, brain, and skeletal muscle. And the adipose tissue *Dicer* knockout mice also showed a decrease of microRNA-450a-5p in adipose tissue and serum. Besides, we found microRNA-450a-5p could impair glucose metabolism by downregulating DUSP10 in liver and adipose tissue. In-*vitro* and in-*vivo* experiments showed that increased microRNA-450a-5p can promote TG synthesis (data not shown), and as the main source of FFAs in vivo, TG can lead to the occurrence of IR and T2DM by inducing inflammatory responses [38, 40]. The candidate target gene of microRNA-450a-5p related to lipid metabolism might be the CREB1, which might regulate lipid metabolism by regulating AKT/mTOR signaling and the CREB1-Bcl2-Beclin1 pathway [49–52]. Besides, Beclin1 is an autophagy marker, and the interplay between CREB1 and Beclin1 can be significant in the context of autophagy and apoptosis [51]. Upregulated autophagy could alleviate IR and T2DM [53, 54], indicating that inhibition of microRNA-450a-5p could enhance the autophagy via CREB1 to alleviate T2DM. It has been reported that over-expressed microRNA-450a-5p reduced the insulin resistance of MGO-induced HUVECs by targeting CREB [31]. Interestingly, over-expressed microRNA-450a-5p could reverse the activity of HUVECs and inhibit cell invasion and fat formation induced by MGO in HUVECs [31]. These findings suggested that microRNA-450a-5p takes different roles in various cells and diseases, and it might be involved in the regulation of proliferative potency of progenitor cells including endothelial progenitor cells and thereby ameliorate their reparative capacity. And the detail function of microRNA-450a-5p in regulation of proliferative potency needs to be further explored. Interestingly, the expression and release of microRNAs can be affected by TG-FFAs-inflammation-ERS in the state of obesity, and whether there is a regulatory interaction between microRNA-450a-5p and TG needs to be further confirmed.

Several studies have shown that microRNA is a potential therapeutic target and biomarker in the tumor, inflammation, and other diseases because of its structural characteristics and easy detection [16, 20, 55, 56]. Constructing a microRNA-mimic/inhibitor equipped with a genetically-engineered vector can achieve targeted gene therapy. For example, nasal inhalation of AAV-microRNA-541 in mice with acute pneumonia can effectively relieve pneumonia symptoms [57]. Moreover, our in-*vitro* and in-*vivo* studies confirmed that the increase in adipose tissue-derived microRNA-450a-5p after obesity could inhibit the JNK/IRS/PI3K/AKT activity via DUSP10 in the liver and adipose tissue; thus, impairing glucose tolerance and promoting the occurrence of IR, and targeted inhibition of microRNA-450a-5p could effectively improve T2DM symptoms. These findings revealed that microRNA-450a-5p has the potential value for the therapy of T2DM and obesity-related diseases.

GA is widely found in palmetacar rhubarb, eucalyptus grandis, dogwood, and other plants, which has anti-inflammatory, anti-oxidation, and anti-tumor effects [58, 59]. The latest study identifies GA’s therapeutic potential in reducing kidney fibrosis, oxidative stress, and inflammation in a glucolipotoxicity-induced diabetic model using db/db mice by downregulating miR-709 [60]. And GA plays a critical role in type I diabetes rats induced by streptozotocin [61], and decreases TG and LDL-C levels in HFD mice [62]. Furthermore, GA could increase autophagy to inhibit tumor [63], and enhancing autophagy activity may be a potential strategy for treating diabetes [54]. These findings indicated GA has a potential therapeutic role in T2DM. Moreover, studies have confirmed that GA can down-regulate the expression of miR-709 [60] and increase the expressions of miR-1247-3p [64], miR-17and miR-92b [65]. These studies have confirmed that GA has the potential to regulate the microRNA content in the body, but whether it can regulate microRNA-450a-5p is not clear. Whether GA has therapeutic effects on obesity and related metabolic diseases via microRNA-450a-5p is unclear. In our study, GA gastric administration in HFD-induced obese mice showed that it could significantly decrease body weight and FBG and improve glucose tolerance and insulin sensitivity. Meanwhile, our study found that GA gastric administration reduced the expression of microRNA-450a-5p in the serum, liver, and adipose tissue of HFD-induced obese mice, suggesting that microRNA-450a-5p may be the target of drug action of GA in vivo. Therefore, exploring whether GA improves glucose metabolism by downregulating microRNA-450a-5p will provide basic experimental data for microRNA-450a-5p as a candidate drug target for the clinical treatment of obesity and related metabolic diseases.

This study acknowledges several limitations that warrant further investigation. Firstly, the mechanisms underlying the elevated expression of microRNA-450a-5p in obesity remain unclear. Future research could benefit from a systematic exploration of factors such as chromatin structure, genomic alterations, and RNA epigenetic modifications. Secondly, while our findings indicate that GA can reduce the levels of microRNA-450a-5p and improve glucose metabolism in obese mice, the precise role of GA in the context of T2DM remains to be elucidated. Specifically, it is necessary to determine whether GA can directly target decrease microRNA-450a-5p to ameliorate T2DM. Addressing these gaps in knowledge could inform the development of early intervention strategies targeting microRNA-450a-5p in obesity, thereby offering valuable insights for the prevention and treatment of T2DM.

## Conclusions

To conclude, our study revealed an important role of adipose tissue-derived microRNA-450a-5p in the occurrence of obesity-associated T2DM. Adipose tissue-derived microRNA-450a-5p downregulated DUSP10 and impaired glucose tolerance and insulin sensitivity, and targeted inhibition of microRNA-450a-5p could improve obesity and T2DM, indicating the functional role of microRNA-450a-5p in obesity and T2DM. Thus, this study elucidated the mechanism and function of adipose tissue-derived microRNA-450a-5p, which will unveil novel research avenues for the treatment of obesity and T2DM (Fig. 8q).

## Materials and methods

### Subjects

From 2019 to 2020, the serum samples of 215 individuals were collected from Xinjiang province of China, including 40 samples from the first affiliated Hospital of Shihezi University, 81 samples from Manas County, and 94 samples from Kashgar. Visceral adipose tissue of individuals (*n* = 12) was collected from the first and the third Affiliated Hospital of Shihezi University. Specific sample sources and grouping criteria can be found in our previous research [66].

### Measurement of biochemical indices

Weight, waist circumference and BMI of all individuals were measured. Blood glucose and lipid levels were detected by automated biochemistry analyzer (Mindray, USA).

### Animal care

Lepr^db/db^, Lepr^db/–^, and C57BL/6 J male mice (4-week-old) were obtained from Changzhou Cavens (Changzhou, China). Dicer^–/–^ and microRNA-450a-5p^–/–^ mice were obtained from Cyagen Biosciences (Guangzhou, China). All animals were of pure C57BL/6 J background. C57BL/6 J mice were fed with HFD for 8 weeks (D12494, 60% energy from fat) according to the criteria defined by Peyot ML49. The mice weighed between 35 and 45 g. The control groups were fed a normal diet (D12450J, 10% energy from fat), and weighed between 25 and 30 g. The experiments adhered to all relevant ethical guidelines and regulations.

### Expression analysis of microRNA-450a-5p

Total RNA in serum samples was extracted using the miRcute serum/plasma microRNA isolation kit (TianGen, DP503) and microRNA-450a-5p in tissues and cells was extracted by TRIZOL (Cat#15596–026, Life Technologies). microRNA-450a-5p cDNA was synthesized from total microRNA using the microRNA first-strand cDNA kit (KR211, TianGen). Next, the relative gene expression was quantified using the microRNA qPCR Kit (FP411, TianGen). The expression is presented as the ratio of the target gene to the internal reference U6.

### RNA isolation and quantitative real-time PCR

Liver, adipose tissue, HepG2, L02, and 3T3-L1 cells were isolated by TRIZOL (Cat#15596–026, Life Technologies). The PCR reaction system consisted of a 10-µL reaction mixture, and real-time PCR assays were performed using QIAGBN (R0315166, QIAGBN). Supplementary Table 1 was used to show the amplified primers.

### Prediction and verification of target genes

Using the bioinformatics database of microRNA target gene prediction such as miRDB (https://mirdb.org/), mirTarbase (https://mirtarbase.cuhk.edu.cn/), and Targetscan (https://www.targetscan.org/), the downstream target genes of microRNA-450a-5p were predicted and analyzed, and a luciferase report assay was performed to identify the indicated target gene. The process is shown in (Fig.S5).

### Western blotting

Total proteins from the tissues and cells were extracted in RIPA Lysis Buffer (Cat#R0010, Solarbio) containing 1% PMSF (Cat#P8340, Solarbio). Western blotting analyses were performed according to standard procedures using indicated antibodies. Supplementary Table 2 was used to list the antibodies.

### Cell culture and transfection

HepG2 and L02 cells were cultured in Dulbecco’s Modified Eagle Medium (DMEM, Gibco), which was supplemented with 10% fetal bovine serum and 1% penicillin/streptomycin (100 µg/mL), and maintained at 37 °C with 5% CO_2_. 3T3-L1 cells were cultured with inducing agents to obtain adipocytes. The hsa-microRNA450a-5p, mmu-microRNA450a-5p mimics, overexpression plasmid of DUSP10, and negative control (NC) were synthesized by Gene Pharma. These constructs were added to cells after they had been incubated with the Lipofectamine 2000 (Catalog#: 11668–019, Invitrogen), adhering to the manufacturer’s protocol.

### In vitro glucose metabolism assay

Cells were grown in 6-well plates with different treatments. The culture medium was collected for measuring glucose concentration using the glucose oxidase method (F006, Nanjing Jiancheng Bioengineering Research Institute). For the insulin sensitivity assay, the 100 nmol/L insulin was used to stimulate cells after different treatments. Culture medium was collected at 15, 30, 45, 60, 90, and 120 min, and glucose concentration was measured.

### Amplification, extraction, and purification of the adenovirus

Human renal epithelial cells of 293A were cultured in a 25 T cell culture flask. When the cells grew to about 50%, the cell culture medium was replaced, the adenovirus stock solution was added to the bottle for 2–3 days, and cells showed grape series change. After cell amplification, the cells were blown from the bottom of the culture bottle, and collecting cell suspension into a 15-mL centrifuge tube centrifuged at 2000 r/min for 10 min, and the supernatant was discarded. DMEM (2–3 mL per 25 T culture vial) was added for cell precipitation which was resuspended using dry ice and subjected to methanol and a 37 °C water bath. Subsequently, it was freeze-thawed for 10 min, three times at 2000 rpm, and centrifuged for 10 min to obtain cell debris precipitate. The supernatant was collected for purification at -80 °C. For specific purification procedures, we referred to the instructions of ViraTrap “M Adenovirus Purification Maxiprep KitViraTrap” adenovirus mass purification kit.

### In vivo glucose-tolerance tests

After determination of FBG levels with 12-h overnight fasting, each mouse received an intraperitoneal bolus of 2 g of glucose per kg of body weight. Blood glucose levels were detected after 15, 30, 45, 60, and 120 min.

### In vivo insulin sensitivity tests

After determination of FBG levels with 6-h fasting, each mouse received an intraperitoneal bolus of 0.5 UI insulin per kg of body weight. Blood glucose levels were detected after 15, 30, 45, 60, and 120 min.

### Statistical analysis

Statistical analyses were conducted using the SPSS 20.0 with unpaired Student’s *t*-test, one-way analysis of variance (ANOVA) and rank-sum test. The relationship between microRNA-450a-5p expression and related clinical metrics was examined using Spearman’s correlation analysis. *P* < 0.05 was considered statistically significant.

## Supplementary Information

Supplementary Material 1.

## Data Availability

No available data and material.
