# Supplementary material for: Adipose tissue-derived microRNA-450a-5p induces type 2 diabetes mellitus by downregulating DUSP10
Source: Mol Biomed. 2025 Feb 6;6:7. doi: 10.1186/s43556-025-00247-w (PMC11803021; doi:10.1186/s43556-025-00247-w)
Supplement: Supplementary file 1 — Supplementary Material 1. [file 43556_2025_247_MOESM1_ESM.zip › General Information of subjects_ESM.pdf]

Adipose tissue-derived microRNA-450a-5p induces type 2 diabetes mellitus by downregulating DUSP10

Running Head: Obesity induced miRNA-450 increasing promotes T2DM

Jiaojiao Zhu[1*], Yanting Hou[1*], Wei Yu[2], Jingzhou Wang[1], Xiaolong Chu[1], Xueting Zhang[1], Huai Pang[1], Dingling Ma[1], Yihan Tang[1], Menghuan Li[1], Chenggang Yuan[1], Jianxin Xie[1#], Cuizhe Wang[1#], Jun Zhang[1#].

1. Medical College of Shihezi University, Bei-Er-Lu, Shihezi 832000 Xinjiang, China.
2. School of Pharmacy, Xinjiang Shihezi University, Xinjiang 832002, China.

J.J.Z and Y.T.H contributed equally to this work.

Correspondence and requests for materials should be addressed to J.X.X. (email: mayue850911@163.com), C.Z.W (email: wangcuizhe905@163.com) or to J.Z. (email: zhangjunyc@163.com)

Number	Age	Sex	Height (cm)	Weight (kg)	BMI	WC (cm)	FPG (mmol/L)	TG (mmol/L)	TC (mmol/L)	LDL-C (mmol/L)	HDL-C (mmol/L)
NC-1	43	male	155	55	22.89281998	67	4.5	1.1	4.92	2.68	1.53
NC-2	43	male	170	66	22.83737024	70	5	0.78	3.43	1.63	1.86
NC-3	43	male	169	65	22.75830678	82	4.1	1.53	4.47	2.18	1.51
NC-4	44	male	150	50	22.22222222	75	4.45	0.87	3.09	2.16	1.37
NC-5	41	male	170	57	19.72318339	77	4.69	0.9	4.26	2.36	1.23
NC-6	43	male	165	53	19.46740129	78	3	0.77	3.81	1.86	1.79
NC-7	43	female	154	54	22.76943835	73	4.3	0.54	4.72	2.38	2.06
NC-8	43	female	149	52	23.42236836	79	4.4	0.86	4.62	2.65	1.48
NC-9	42	female	153	54	23.06805075	74	5.32	0.74	3.13	1.2	3.8
NC-10	43	female	158	48	19.22768787	81	4.6	1.13	2.17	1.16	1.11
NC-11	43	female	155	57	23.72528616	82	4.3	1.23	5.1	2.52	2.1
NC-12	43	female	164	56	20.82093992	83	5.9	1.57	4.59	2.42	1.92
NC-13	40	male	167	62	22.23098713	82	4.9	1.05	4.12	2.29	1.45
NC-14	41	male	170	65	22.49134948	84	5.1	0.81	3.93	2.14	1.09
NC-15	41	male	170	63	21.79930796	85	5.7	1.61	4.41	2.38	1.24
NC-16	46	male	172	62	20.9572742	67	4.7	0.61	4.85	2.32	2.16
NC-17	46	male	165	60	22.03856749	80	6	0.87	4.24	1.74	2.16
NC-18	47	male	170	65	22.49134948	85	4.1	0.78	4.67	2.44	1.93
NC-19	40	female	158	56	22.43230252	80	5.2	0.79	3.8	1.88	1.6
NC-20	41	female	168	55	19.48696145	74	4.1	1.31	4.13	1.8	2.24
NC-21	41	female	151	52	22.80601728	82	4.4	0.8	4.24	1.99	1.94
NC-22	46	female	158	58	23.23345618	82	4.7	1.02	4.12	2.17	2.15
NC-23	46	female	150	53	23.55555556	80	4.4	0.84	4.48	2.24	2.25
NC-24	47	female	154	47	19.81784449	81	3.68	0.48	4.15	1.25	2.44
NC-25	43	male	171	68	23.25501864	85	4.51	0.9	4.54	2.42	1.48
NC-26	43	male	178	73	23.0400202	83	3.69	0.37	3.83	1.65	1.04
NC-27	43	male	179	66	20.59860803	73	5.24	0.37	4.53	1.96	1.47
NC-28	44	male	171	65	22.22906193	68	4.8	0.7	4.61	2.13	1.65
NC-29	42	male	170	58	20.06920415	70	3.86	0.81	4.9	2.34	1.29
NC-30	43	female	160	61	23.828125	84	3.8	0.89	5.09	2.43	1.42
NC-31	43	female	161	56	21.60410478	80	4.87	0.44	4.02	1.51	1.59
NC-32	43	female	161	55	21.21831719	78	4.95	1.08	4.49	1.87	1.86
NC-33	43	female	167	63	22.58955144	74	3.97	1.02	5.12	2.19	1.85
NC-34	43	female	155	48	19.97918835	67	4.29	0.83	4.67	2.06	1.83
NC-35	42	female	160	55	21.484375	68	3.88	0.39	4.54	1.96	1.6
NC-36	42	male	170	62	21.4532872	85	3.26	0.8	4.19	2.15	1.4
NC-37	42	male	168	63	22.32142857	72	3.97	0.64	4.52	2.34	1.54
NC-38	44	male	180	75	23.14814815	80	4.09	0.37	4.24	1.88	1.65
NC-39	44	male	179	71	22.15910864	80	3.52	0.84	5.05	2.14	1.63
NC-40	44	male	166	65	23.58832922	80	4.22	0.4	4.28	2.24	0.79
NC-41	44	male	169	59	20.65754	70	4.31	0.73	4.01	1.42	1.31
NC-42	42	female	165	56	20.56932966	80	4.68	0.38	4.27	1.45	1.53
NC-43	42	female	165	52	19.10009183	73	3.8	0.43	5.03	2.33	1.56
NC-44	44	female	156	56	23.01117686	85	3.98	0.56	4.6	3.3	1.74
NC-45	44	female	160	61	23.828125	80	3.51	0.87	4.79	2.57	0.54
NC-46	44	female	164	63	23.42355741	78	4.1	0.77	3.46	1.6	1.49
NC-47	44	female	157	50	20.28479857	70	4.18	0.73	4.74	2	0.95
NC-48	45	male	184	70	20.6758034	92	5.31	1.5	4.8	2.72	1.03
NC-49	44	male	175	68	22.20408163	88	4.78	1.12	3.06	1.49	0.98
NC-50	44	female	165	60	22.03856749	81	5.06	1.45	6.66		
NC-51	43	female	158	50	20.02884153	77	5.56	1.13	4.17		
NC-52	40	female	163	52	19.57168128	80	5.52	0.65	5.82		
NC-53	50	female	166	58	21.04804761	76	3.37	0.54	4.64	2.23	1.74
NC-54	45	female	161	60	23.14725512	84	5.79	1.51	4.5	2.41	1.2
NC-55	43	male	168	58	20.54988662	75	5.24	1.12	4.83		
NC-56	52	male	175	62	20.24489796	80	4.65	1.3	5.7	3.13	1.66
NC-57	50	male	166	61	22.13673973	83	5.53	1.58	6.06	4.12	1.25
NC-58	41	female	156	55	22.60026298	74	4.48	1.53	4.03		
NC-59	48	male	165	65	23.87511478	84	4.17	0.62	4.19	2.33	1.31
NC-60	47	male	170	67	23.183391	91	4.7	0.65	3.83	1.97	1.41

obesity-1	40	male	173	97	32.41003709	111	5.1	0.87	4.4	2.1	1.83
obesity-2	40	male	171	90	30.77870114	107	5.9	1.02	5.11	3.02	1.7
obesity-3	40	male	168	89	31.53344671	109	4.9	0.74	3.69	1.75	2.9
obesity-4	41	male	157	92	37.32402937	108	5	1.38	4.9	2.55	2
obesity-5	41	male	166	92	33.38655828	109	4.5	1.28	3.91	1.43	2.15
obesity-6	44	male	164	80	29.74419988	106	5.8	1.35	4.05	2.11	1.65
obesity-7	41	female	156	84	34.51676529	112	4.37	1.31	4.21	2.11	1.12
obesity-8	41	female	158	90	36.05191476	109	5.5	1.39	4.99	2.69	1.98
obesity-9	42	female	152	76	32.89473684	113	4.2	1.06	5.07	2.11	2.04
obesity-10	42	female	154	84	35.41912633	101	4.1	0.86	4.19	2.15	1.46
obesity-11	43	female	152	82	35.49168975	106	5.05	1.11	4.41	2.03	2.15
obesity-12	43	female	160	74	28.90625	104	4.4	1.16	4.53	2.45	2.11
obesity-13	45	male	170	98	33.9100346	117	5.7	1.47	4.32	2.36	1.42
obesity-14	45	male	173	97	32.41003709	114	4.8	1.6	4.05	2.25	1.35
obesity-15	45	male	168	80	28.3446712	103	6	1.68	5.08	2.91	1.53
obesity-16	46	male	175	90	29.3877551	104	5.9	1.36	4.12	2.11	1.65
obesity-17	47	male	162	83	31.62627648	102	5.9	0.86	4.85	2.54	2.2
obesity-18	47	male	163	97	36.50871316	119	5.3	0.63	4.92	2.75	1.93
obesity-19	48	female	152	81	35.05886427	108	3.25	1.23	3.66	2.3	3.21
obesity-20	45	female	160	93	36.328125	105	5.1	1.67	4.58	2.25	1.97
obesity-21	45	female	150	73	32.44444444	102	4.8	0.85	5.03	1.6	2.7
obesity-22	46	female	154	94	39.63568899	102	5.09	1.04	4.69	2.36	1.23
obesity-23	47	female	155	74	30.8012487	101	4.6	1.47	5.14	2.77	2.04
obesity-24	48	female	140	70	35.71428571	117	5.4	1.36	4.43	2.26	1.69
obesity-25	43	male	171	82	28.04281659	85	6.1	4.1	8.24	4.61	2.32
obesity-26	43	male	165	85	31.22130395	87	3.71	1.97	6.55		
obesity-27	43	male	150	80	35.55555556	60	3.64	1.79	2.79		
obesity-28	43	male	158	70	28.04037814	89	3.83	1.19	3.95		
obesity-29	44	male	160	97	37.890625	76	2.85	3.05	4.39		
obesity-30	44	male	155	70	29.13631634	60	5.1	1.36	4.82		
obesity-31	43	female	151	65	28.5075216	84	5.8	1.86	4.57	2	
obesity-32	43	female	157	81	32.86137369	84	5.5	0.79	3.89	1.92	1.82
obesity-33	43	female	153	66	28.19428425	83	4.41	0.7	4.96	3.4	1.3
obesity-34	43	female	147	72	33.31945023	83	5.1	1.12	5.13	2.46	2.36
obesity-35	44	female	151	69	30.26183062	80	4.16	1.3	4.19		
obesity-36	44	female	157	79	32.04998174	79	4.61	0.35	4.56		
obesity-37	40	male	161	75	28.9340689	86	3.91	2.36	5.8	3.56	1.16
obesity-38	41	male	150	65	28.88888889	83	3.53	0.69	5.21		
obesity-39	42	male	160	78	30.46875	80	5	1.25	4.64	2.52	1.7
obesity-40	45	male	161	79	30.47721924	89	5.9	1.93	6.92	4.21	2.22
obesity-41	49	male	160	80	31.25	63	4.12	1.19	4.83		
obesity-42	40	female	163	76	28.60476495	77	5.7	1.11	7.22	4.1	2.94
obesity-43	41	female	160	73	28.515625	84	4.48	0.63	4.66		
obesity-44	42	female	156	77	31.64036818	83	5.09	4.29	5.09		
obesity-45	45	female	150	79	35.11111111	83	4.8	0.79	5.47	2.52	2.57
obesity-46	46	female	155	68	28.30385016	43	4.16	1.44	6.4		2.15
obesity-47	40	male	179	97	30.27371181	100	5.06	1.36	4.19	2.36	1.16
obesity-48	41	male	165	84	30.85399449	96	3.98	0.62	4.89	2.37	1.64
obesity-49	41	male	165	80	29.38475666	95	4.75	0.85	4.93	3.28	1.24
obesity-50	42	male	177	98	31.28092183	97	5.29	1.53	5.14	3.13	1.11
obesity-51	43	male	170	92	31.83391003	108	4.31	0.52	4.62	2.5	1.14
obesity-52	44	male	170	87	30.10380623	93	4.22	1.08	4.25	2.25	1.22
obesity-53	41	female	162	86	32.76939491	115	4.56	0.8	4.79	2.39	1.61
obesity-54	41	female	159	73	28.87544005	98	3.07	0.73	4.15	2.06	1.57
obesity-55	42	female	166	90	32.66076354	105	4.05	1.18	3.52	1.55	1.38
obesity-56	42	female	167	98	35.13930223	97	3.93	1.24	4.75	2.6	1.53
obesity-57	43	female	168	98	34.72222222	100	3.86	1.07	4.88	2.39	1.31
obesity-58	43	female	153	73	31.18458712	100	3.11	0.58	3.95	1.53	1.76
obesity-59	42	male	175	88	28.73469388	100	4.14	1.49	5.01	3.44	1.17
obesity-60	42	male	169	84	29.41073492	97	3.91	0.41	3.59	1.42	1.53
obesity-61	43	male	154	88	37.10575139	98	3.97	1.36	4.95	2.45	1.1

obesity-62	45	male	173	98	32.74416118	110	3.85	0.63	4.56	2.95	1.75
obesity-63	46	male	176	91	29.37758264	98	4.61	0.62	4.68	2.34	1.49
obesity-64	47	male	169	86	30.11099051	95	4.97	0.71	2.97	1.29	1.07
obesity-65	42	female	160	80	31.25	94	3.52	0.48	4.65	1.98	1.57
obesity-66	43	female	167	82	29.4022733	94	4.33	0.65	3.94	1.8	1.42
obesity-67	43	female	159	79	31.24876389	90	4.12	0.95	5.16	2.58	1.98
obesity-68	45	female	161	97	37.42139578	101	3.83	0.55	3.82	1.62	1.48
obesity-69	46	female	156	72	29.58579882	90	4.24	0.98	5.05	2.77	1.56
obesity-70	47	female	157	78	31.64428577	100	3.47	0.38	4.98	1.93	1.44
obesity-71	41	male	164	76	28.25698989	84	3.92	1.04	5.08	3.13	1.19
obesity-72	41	male	161	75	28.9340689	88	4.8	3.76	5.54	2.68	1.44
obesity-73	43	male	168	81	28.69897959	85	4.07	1.41	6.17	3.58	1.13
obesity-74	46	male	174	87	28.73563218	80	4.01	3.05	8.05	4.68	1.73
obesity-75	47	male	161	76	29.31985649	88	4.32	0.66	6.84	3.9	1.77
obesity-76	48	male	153	73	31.18458712	88	4.04	0.73	7.33	3.41	1.26
obesity-77	41	female	156	71	29.17488494	84	4.09	0.65	5.4	4.35	1.94
obesity-78	41	female	160	75	29.296875	83	3.99	1.39	6.87	3.35	2.38
obesity-79	42	female	152	65	28.13365651	80	4.02	0.86	4.4	2.82	1.25
obesity-80	47	female	150	67	29.77777778	82	3.25	1.02	4.26	1.83	1.17
obesity-81	49	female	159	81	32.03987184	84	4.52	1.32	5.7	4.91	1.74
obesity-82	52	female	158	70	28.04037814	80	4.11	0.79	5.16	1.94	1.04
obesity-83	26	male	161	74	28.54828132	87	3.43	2.91	6.06	3.48	1.29
obesity-84	29	male	168	85	30.11621315	82	3.68	2.2	5.94	3.13	1.69
obesity-85	29	male	168	88	31.17913832	82	3.68	2.2	5.94	3.13	1.69
obesity-86	33	male	164	76	28.25698989	82	4.72	0.94	4.49	1.94	1.68
obesity-87	35	male	169	80	28.01022373	85	4.23	1.44	5.33	3.38	1.54
obesity-88	62	male	151	72	31.57756239	87	4.51	0.81	5.76	2.65	1.84
obesity-89	27	female	153	69	29.47584262	84	3.28	0.64	4.53	1.9	1.04
obesity-90	28	female	167	80	28.68514468	83	4.14	0.43	5.92	2.52	1.09
obesity-91	33	female	158	71	28.44095498	84	4.11	0.84	5.9	2.79	2.07
obesity-92	34	female	168	81	28.69897959	78	3.45	0.93	5.04	3.02	1.36
obesity-93	35	female	160	76	29.6875	80	3.24	0.74	4.59	2.47	1.48
obesity-94	59	female	139	60	31.05429326	84	4.26	0.93	5.66	2.87	1.63
obesity-95	40	male	170	88	30.44982699	102	4.6	1.49	3.64	2.06	1.07
obesity-96	50	male	180	95	29.32098765	112	5.44	2.08	5.6	3.23	1.36
obesity-97	46	male	184	95	28.0600189	103	4.88	2.09	6.08	3.99	1.3
obesity-98	43	male	173	86	28.73467206	104	5.11	3.28	4.97	3.22	0.86
obesity-99	46	male	170	88	30.44982699	98	4.97	3.25	5.21	3.11	1.07
obesity-100	50	female	159	72	28.47988608	93	4.57	1.56	3.81	2.01	1.15
obesity-101	49	female	160	73	28.515625	106	5.16	1.58	4.74	2.76	1.29
obesity-102	52	female	160	80	31.25	108	4.09	2.32	4.4	2.25	1.21
obesity-103	42	male	168	87	30.82482993	105	5.07	8.41	4.65		
obesity-104	45	male	178	93	29.3523545	106	5.22	2.42	4.92	2.59	1.26
obesity-105	47	male	177	90	28.72737719	97	5.96	2.08	3.69	2	1.06
T2DM-1	43	male	179	105	32.77051278	110	8.5	6.04	7.29	1.98	4.14
T2DM-2	43	male	162	86	32.76939491	104	13.7	3.52	6.76	3.84	2.08
T2DM-3	42	male	167	81	29.04370899	100	8.2	4.62	5.98	3.17	2
T2DM-4	42	male	162	93	35.43667124	111	7.01	7.33	9.88	1.23	3.25
T2DM-5	42	male	165	86	31.58861341	106	13.9	6.53	6.64	3.64	1.75
T2DM-6	42	male	151	78	34.20902592	105	7.2	4.57	5.79	3.25	1.31
T2DM-7	42	female	158	78	31.24499279	110	7	3.47	5.53	3.05	2.12
T2DM-8	44	female	159	97	38.36873541	120	7	2.18	5.74	3.02	1.67
T2DM-9	44	female	154	69	29.09428234	97	8.1	3.37	7.57	4.16	3.07
T2DM-10	45	female	153	72	30.757401	99	11.7	4.63	5.92	3.24	2.02
T2DM-11	46	female	150	70	31.11111111	99	7.26	4.52	5.54	1.69	3.23
T2DM-12	46	female	154	73	30.7809074	112	7.2	8.15	7.29	3.91	2.14
T2DM-13	46	male	162	81	30.86419753	99	7	5.15	8.78	5.06	2.18
T2DM-14	48	male	172	87	29.40778799	103	8.6	5.2	5.35	3.01	1.37
T2DM-15	49	male	168	92	32.59637188	102	7.1	4.48	5.51	3.1	1.62
T2DM-16	49	male	183	110	32.84660635	117	9.01	4	5.82	3.6	1.21
T2DM-17	49	male	173	94	31.40766481	107	14.8	2.4	8.03	4.3	2.38

T2DM-18	49	male	165	94	34.52708907	106	7.1	4	6.82	3.88	1.76
T2DM-19	47	female	153	71	30.33021487	106	7.6	3.02	5.84	1.81	3.08
T2DM-20	48	female	157	76	30.83289383	104	7	4.42	6.09	3.55	1.73
T2DM-21	49	female	155	70	29.13631634	118	7.2	3.29	6.23	2.99	2.23
T2DM-22	49	female	158	99	39.65710623	111	8	2.66	6.25	3.45	1.84
T2DM-23	50	female	161	91	35.10667027	118	7.5	1.72	6.92	3.19	2.72
T2DM-24	50	female	165	97	35.62901745	117	8.5	4.14	5.41	2.91	1.9
T2DM-25	42	female	165	67	24.6097337	82	10.22	0.19	4.5	1.7	2.69
T2DM-26	62	female	162	72	27.43484225	90	7.14	2.54	6.33	3.48	1.39
T2DM-27	54	female	154	64	26.98600101	77	18.79	1.1	5.13	2.12	2.22
T2DM-28	42	female	164	57	21.19274242	77	12.13	1.11	4.8	2.69	0.72
T2DM-29	41	female	161	58	22.37567995	78	10.73	0.96	5.73	3.47	1.84
T2DM-30	57	female	166	78	28.30599506	90	8.96	1.74	6.42	3.87	1.52
T2DM-31	56	female	145	53	25.20808561	82	20.34	8.42	11.53	6.01	1.42
T2DM-32	54	male	171	71	24.28097534	75	7.92	0.72	4.59	2	1.73
T2DM-33	42	male	182	102	30.79338244	93	16.52	1.6	8.32	5.26	1.46
T2DM-34	57	male	176	96	30.99173554	95	12.8	7.03	6.2	3.14	1.45
T2DM-35	63	female	154	60	25.29937595	93	10.97	3.6	6.18	3.47	1.71
T2DM-36	58	female	175	72	23.51020408	92	10.1	1.74	5.02	3.09	1.16
T2DM-37	44	female	158	58	23.23345618	85	10.45	2.52	4.86	2.52	1.22
T2DM-38	43	female	163	94	35.3795777	115	7.08	0.92	3.84	2.05	1.25
T2DM-39	53	female	161	58	22.37567995	81	12.57	2.38	5.12	2.8	1.37
T2DM-40	56	female	156	63	25.88757396	94	9.64	1.31	4.2	2.29	1.2
T2DM-41	56	female	166	65	23.58832922	93	7.14	0.97	2.93	1.45	1.1
T2DM-42	68	female	158	91	36.45249159	118	8.4	1.29	3.56	2.04	0.99
T2DM-43	45	female					12.11	0.75	4.3	2.06	1.58
T2DM-44	65	male	170	60	20.76124567	83	9.56	0.69	4.07	2.28	1.33
T2DM-45	53	male	170	66	22.83737024	85	8.1	1.82	4.48	3.42	0.75
T2DM-46	31	male	171	76	25.99090318	92	7.21	1.57	5.47	4.12	1.49
T2DM-47	54	male	170	68	23.52941176	85	10.85	0.84	3.9	2.12	1.23
T2DM-48	47	male	174	92	30.3871053	98	7.81	5.61	5.14	2.62	1.51
T2DM-49	53	male	185	98	28.63403944	103	8.06	3.54	3.48	1.63	0.93
T2DM-50	42	male	165	64	23.50780533	84	14.58	1.78	5.41	3.55	1.18
