# Supplementary material for: Adipose tissue-derived microRNA-450a-5p induces type 2 diabetes mellitus by downregulating DUSP10
Source: Mol Biomed. 2025 Feb 6;6:7. doi: 10.1186/s43556-025-00247-w (PMC11803021; doi:10.1186/s43556-025-00247-w)
Supplement: Supplementary file 1 — Supplementary Material 1. [file 43556_2025_247_MOESM1_ESM.zip › supplementary files_ESM.pdf]

Adipose tissue-derived microRNA-450a-5p induces type 2 diabetes mellitus by downregulating DUSP10

Running Head: Obesity induced miRNA-450 increasing promotes T2DM

Jiaojiao Zhu[1*], Yanting Hou[1*], Wei Yu[2], Jingzhou Wang[1], Xiaolong Chu[1], Xueting Zhang[1], Huai Pang[1], Dingling Ma[1], Yihan Tang[1], Menghuan Li[1], Chenggang Yuan[1], Jianxin Xie[1#], Cuizhe Wang[1#], Jun Zhang[1#].

1. Medical College of Shihezi University, Bei-Er-Lu, Shihezi 832000 Xinjiang, China.

2. School of Pharmacy, Xinjiang Shihezi University, Xinjiang 832002, China.

J.J.Z and Y.T.H contributed equally to this work.

Correspondence and requests for materials should be addressed to J.X.X. (email: mayue850911@163.com), C.Z.W (email: wangcuizhe905@163.com) or to J.Z. (email: zhangjunyc@163.com)

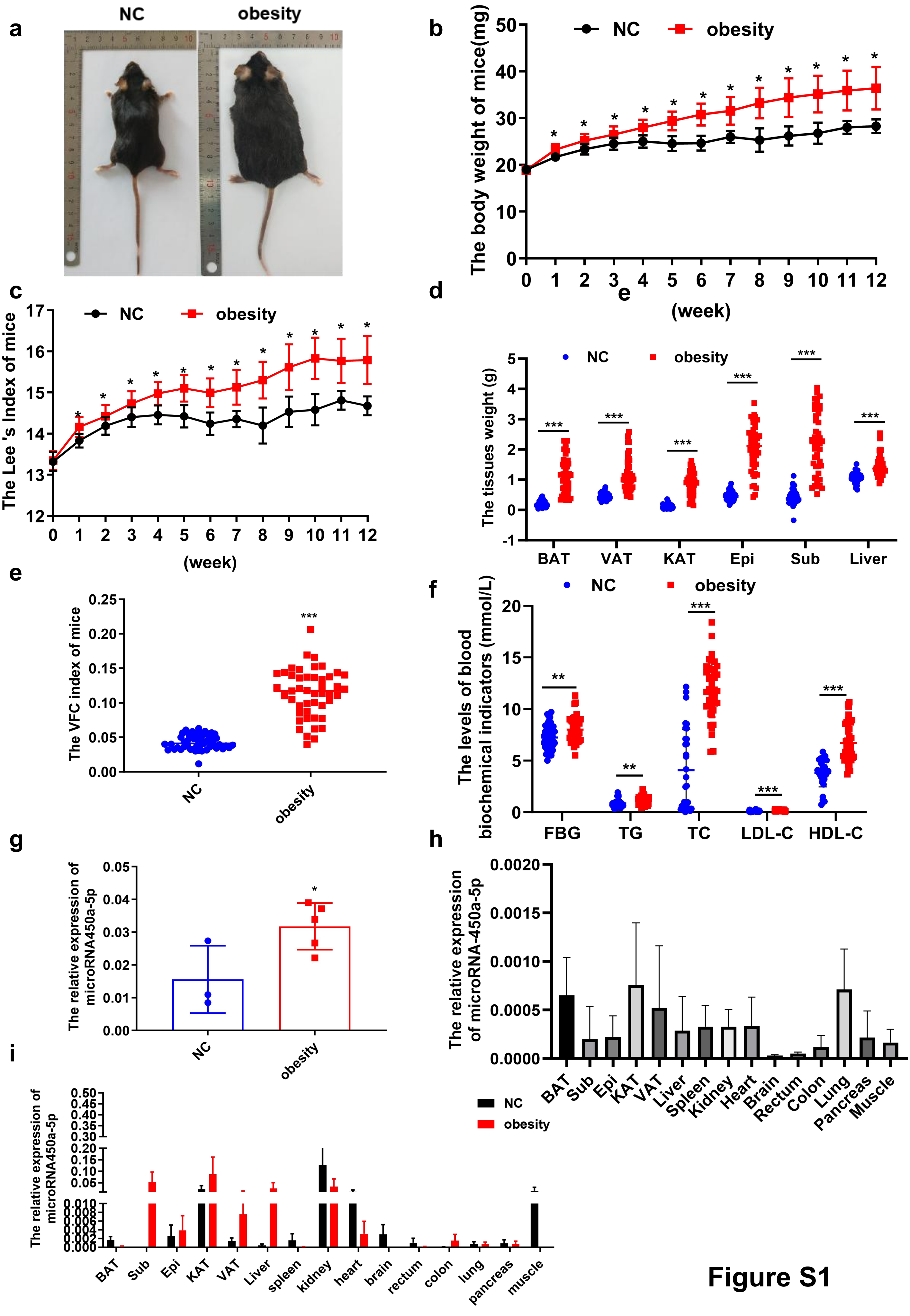
a
NC
obesity
b
The body weight of mice(mg)
NC
obesity
(week)
c
The Lee 's Index of mice
NC
obesity
(week)
d
The tissues weight (g)
NC
obesity
BAT
VAT
KAT
Epi
Sub
Liver
e
The VFC index of mice
NC
obesity
f
The levels of blood biochemical indicators (mmol/L)
NC
obesity
FBG
TG
TC
LDL-C
HDL-C
g
The relative expression of microRNA450a-5p
NC
obesity
h
The relative expression of microRNA-450a-5p
BAT
Sub
Epi
KAT
VAT
Liver
Spleen
Kidney
Heart
Brain
Rectum
Colon
Lung
Pancreas
Muscle
i
The relative expression of microRNA450a-5p
NC
obesity
BAT
Sub
Epi
KAT
VAT
Liver
spleen
kidney
heart
brain
rectum
colon
lung
pancreas
muscle

Figure S1

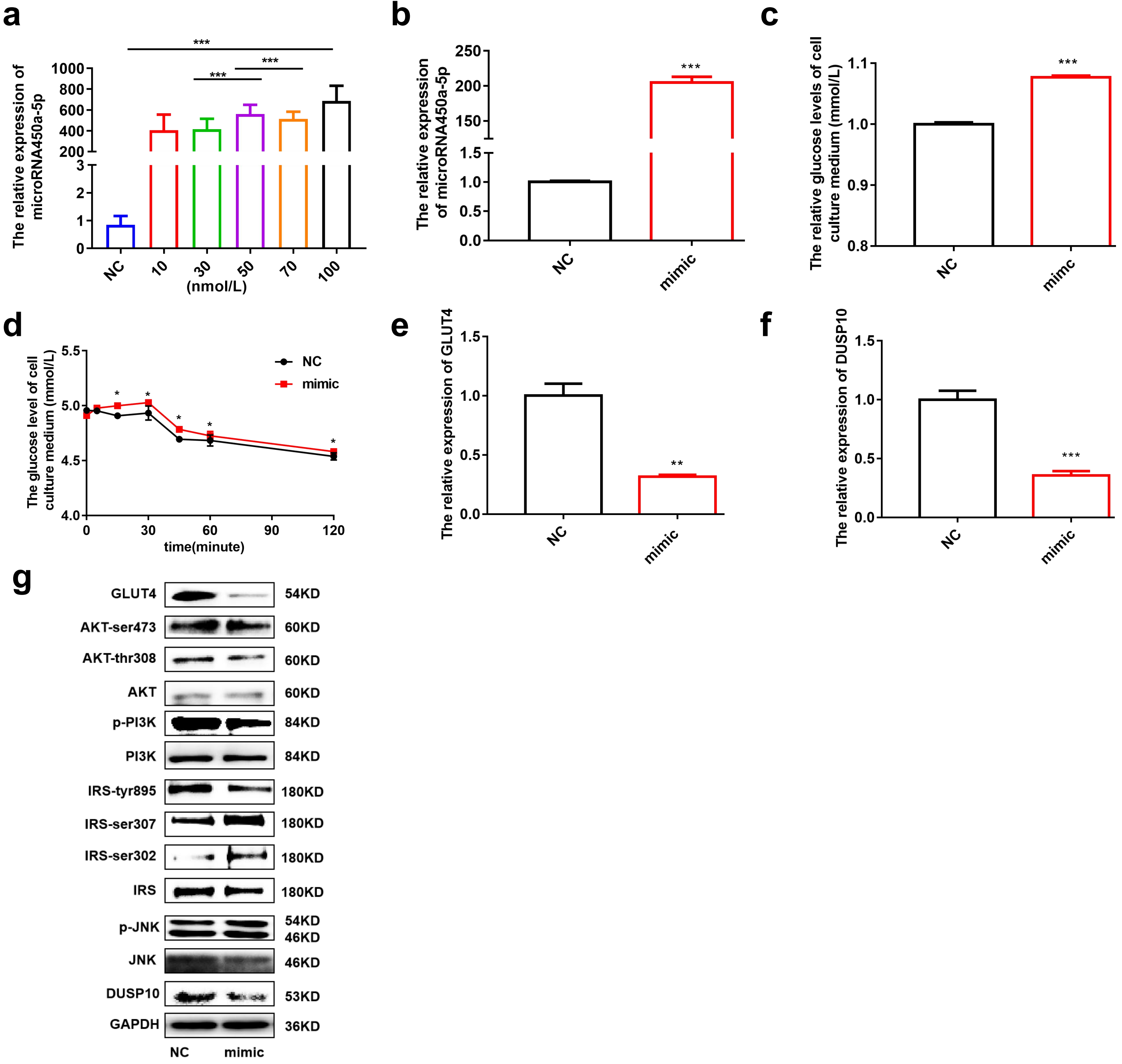

a
The relative expression of microRNA450a-5p
1000
800
600
400
200
3
2
1
0
***
***
***
NC
10
30
50
70
100
(nmol/L)
b
The relative expression of microRNA450a-5p
250
200
150
1.5
1.0
0.5
0.0
***
NC
mimic
c
The relative glucose levels of cell culture medium (mmol/L)
1.1
1.0
0.9
0.8
***
NC
mimc
d
The glucose level of cell culture medium (mmol/L)
5.5
5.0
4.5
4.0
0
30
60
90
120
time(minute)
NC
mimic
e
The relative expression of GLUT4
1.5
1.0
0.5
0.0
**
NC
mimic
f
The relative expression of DUSP10
1.5
1.0
0.5
0.0
***
NC
mimic
g
GLUT4 54KD
AKT-ser473 60KD
AKT-thr308 60KD
AKT 60KD
p-PI3K 84KD
PI3K 84KD
IRS-tyr895 180KD
IRS-ser307 180KD
IRS-ser302 180KD
IRS 180KD
p-JNK 54KD 46KD
JNK 46KD
DUSP10 53KD
GAPDH 36KD
NC
mimic

Figure S2

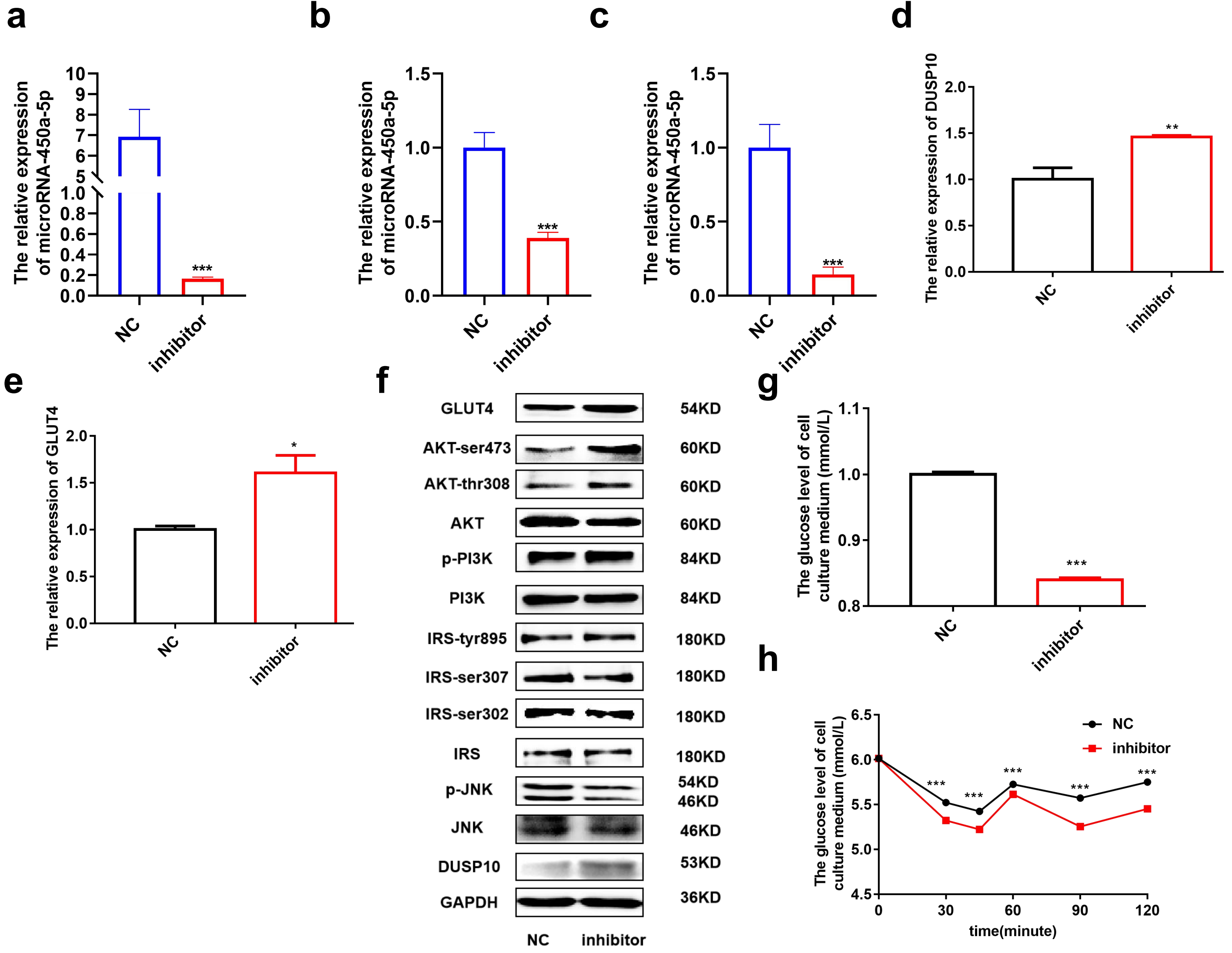
a
b
c
d
e
f
g
h
The relative expression of microRNA-450a-5p
The relative expression of DUSP10
The relative expression of GLUT4
The glucose level of cell culture medium (mmol/L)
NC
inhibitor
***
**
*
GLUT4 54KD
AKT-ser473 60KD
AKT-thr308 60KD
AKT 60KD
p-PI3K 84KD
PI3K 84KD
IRS-tyr895 180KD
IRS-ser307 180KD
IRS-ser302 180KD
IRS 180KD
p-JNK 54KD 46KD
JNK 46KD
DUSP10 53KD
GAPDH 36KD
time(minute)

Figure S3

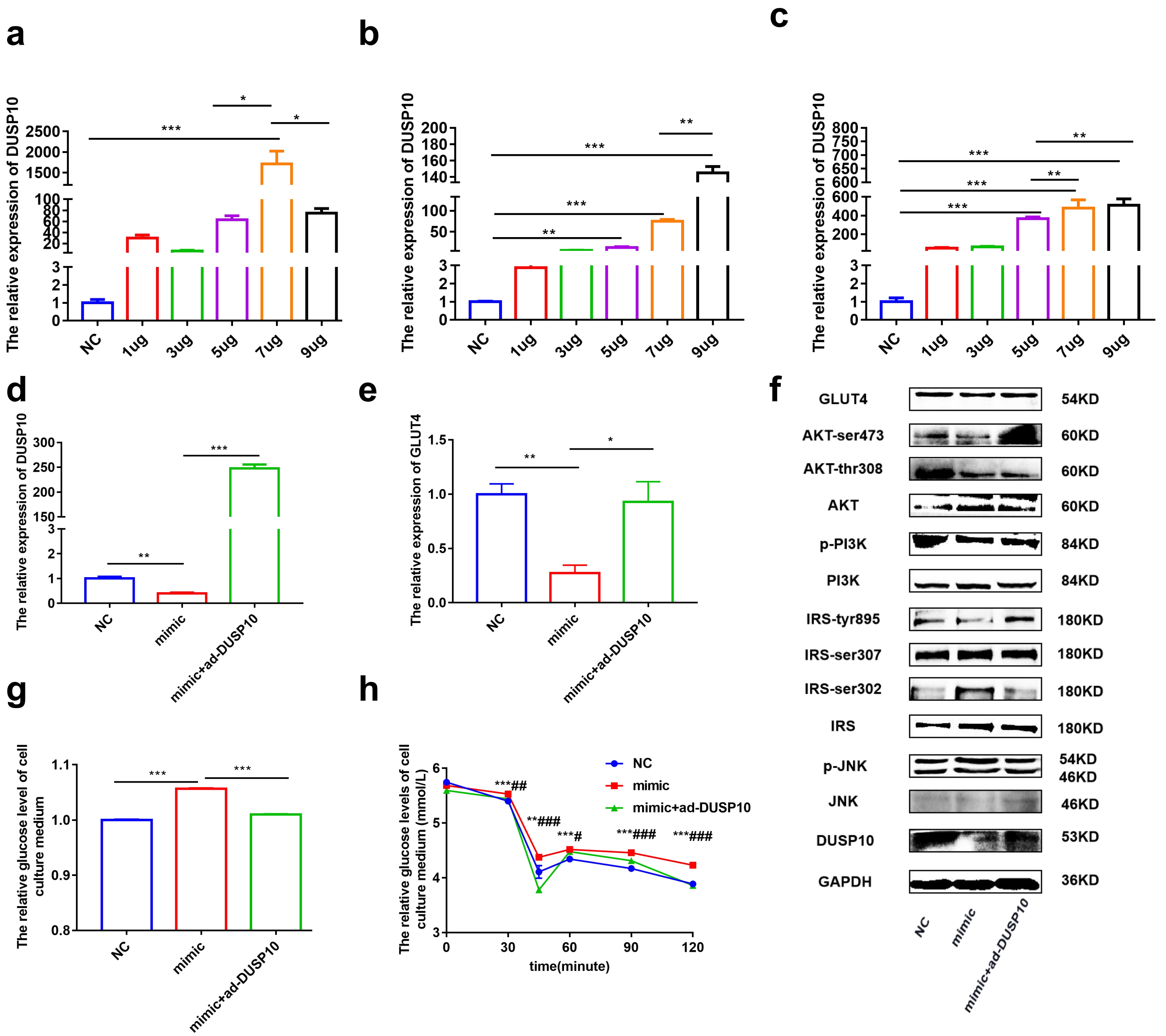
a
b
c
d
e
f
g
h
The relative expression of DUSP10
NC
1ug
3ug
5ug
7ug
9ug
mimic
mimic+ad-DUSP10
The relative expression of GLUT4
GLUT4
54KD
AKT-ser473
60KD
AKT-thr308
60KD
AKT
60KD
p-PI3K
84KD
PI3K
84KD
IRS-tyr895
180KD
IRS-ser307
180KD
IRS-ser302
180KD
IRS
180KD
p-JNK
54KD
46KD
JNK
46KD
DUSP10
53KD
GAPDH
36KD
The relative glucose level of cell culture medium
The relative glucose levels of cell culture medium (mmol/L)
time(minute)
***##
**###
***#
***###
***###

Figure S4

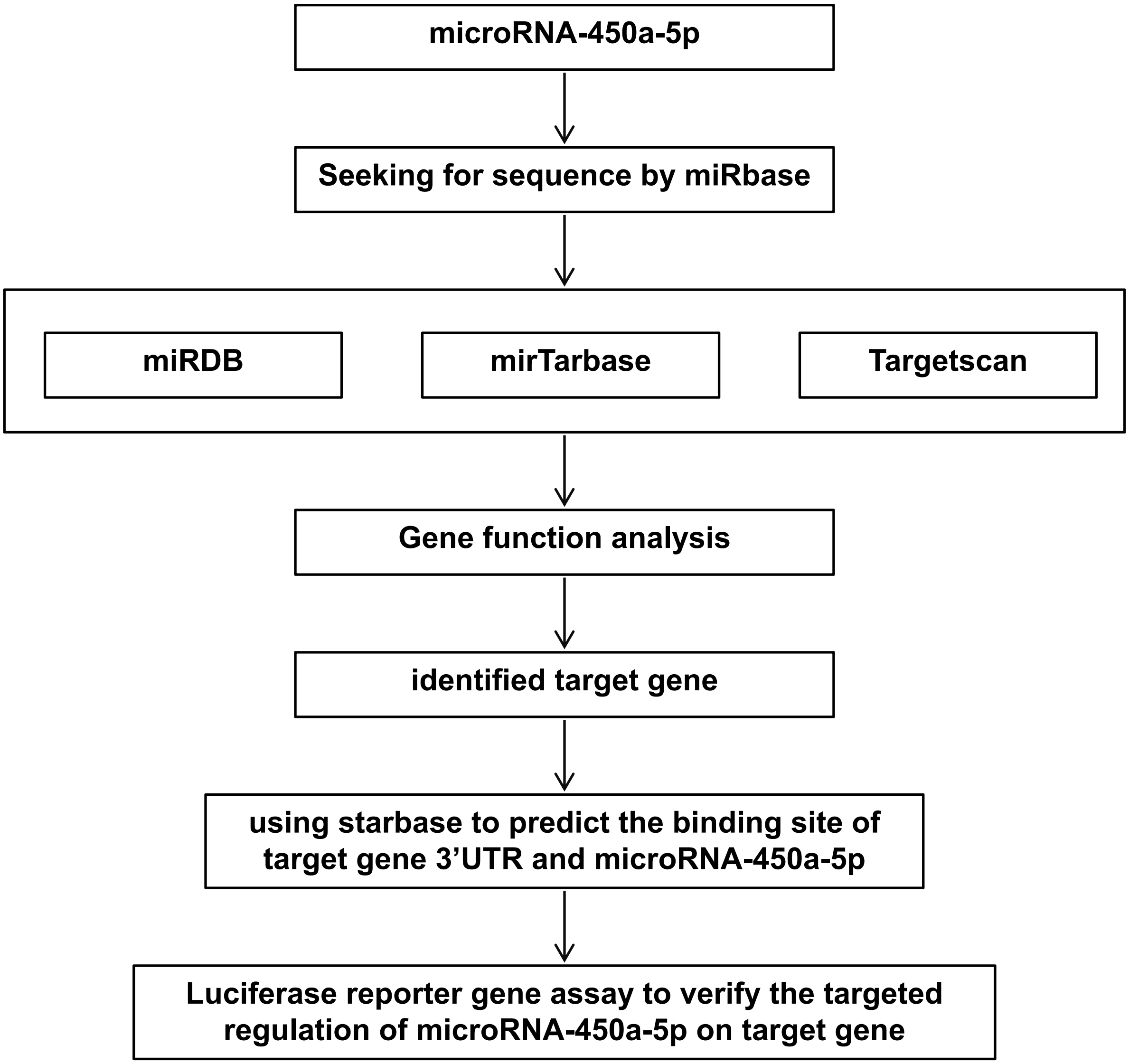
microRNA-450a-5p
Seeking for sequence by miRbase
miRDB
mirTarbase
Targetscan
Gene function analysis
identified target gene
using starbase to predict the binding site of target gene 3'UTR and microRNA-450a-5p
Luciferase reporter gene assay to verify the targeted regulation of microRNA-450a-5p on target gene

Figure S5

Supplementary figure legends

Figure S1. microRNA-450a-5p levels significantly increased in obesity mice.

(**a**), the general picture of the mice at sacrifice. (**b**), the body weight. (**c**), the Lee`s index. (**d**), the weight of brown adipose tissue (BAT), visceral adipose tissue (VAT), Perirenal adipose tissue (KAT), epididymal white adipose tissue (Epi), Subcutaneous adipose tissue (Sub) and Liver. (**e**), the VCF index. (**f**), the serum levels of fast blood (FBG), triglyceride (TG) levels, cholesterol (TC) levels, Low-Density Lipoprotein Cholesterol (LDL-C) levels and Low-Density Lipoprotein Cholesterol (HDL-C) levels. (**g**), the serum microRNA-450a-5p levels. (**h**), microRNA-450a-5p levels in different tissues. (**i**), tissues microRNA-450a-5p levels. Normal diet group (NC), n=6; high fat diet group (obesity), n=6. The *p*-values by two-tailed unpaired Student's *t* test are indicated, Data represent the mean±*SD*, $^{*}p<0.05$ means a statistically significant difference.

Figure S2. microRNA-450a-5p impairs glucose metabolism.

(**a**), the expression level of microRNA-450a-5 in cells stimulated by different concentrations of mimic. After over-expression of microRNA-450a-5p in L02 cells, (**b**), the expression level of microRNA-450a-5 in cells stimulated by 50 nM mimic; (**c**), the glucose consumption; (**d**), the insulin sensitivity; (**e**), the mRNA expression level of DUSP10; (**f**), the mRNA expression level of GLUT4; (**g**), the protein expression of DUSP10/JNK/IRS/PI3K/AKT/GLUT4. The *p*-values by one-way ANOVA in **a**; The *p*-values by two-tailed unpaired Student's *t* test are indicated in **b-f**, $^{*}p<0.05$ means a statistically significant difference.

Figure S3. Inhibition of microRNA-450a-5p promotes glucose metabolism.

The inhibitor of microRNA-450a-5p inhibits the expression of microRNA-450a-5p, (**a**), in HepG2 cells, (**b**), in 3T3-L1 cells, (**c**), in L02 cells. After inhibition of microRNA-450a-5p in L02 cells, (**d**), the mRNA expression level of DUSP10; (**e**), the mRNA expression level of GLUT4; (**f**), the protein expression of DUSP10/JNK/IRS/PI3K/AKT/GLUT4; (**g**), the glucose consumption; (**h**), the insulin sensitivity. The *p*-values by two-tailed unpaired Student's *t* test are indicated, $^{*}p<0.05$ means a statistically significant difference.

Figure S4. microRNA-450a-5p impairs glucose metabolism via downregulating DUSP10.

Overexpression of microRNA-450a-5p with DUSP10 upregulation by plasmid, the mRNA expression level of DUSP10 with different concentrations DUSP10 over-expression plasmid, (**a**) in HepG2 cells, (**b**) in 3T3-L1 cells, (**c**) in L02 cells; (**d**), the mRNA expression level of DUSP10 in L02 cells; (**e**), the mRNA expression level of GLUT4 in L02 cells; (**e**), the protein expression of DUSP10/JNK/IRS/PI3K/AKT/GLUT4; (**f**), the glucose consumption; (**g**), the insulin sensitivity. The *p*-values by one-way ANOVA are indicated, data represent the mean±*SD*. Compared with NC group, $^{*}p<0.05$ means a statistically significant difference, compared with mimic group, $^{\#}p<0.05$ means a statistically significant difference.

Figure S5. The process of target genes predicted and verified.

Supplementary tables

Supplementary Table 1 The amplified primer's sequence of PCR

	**Primer**	**Sequence (5'→3')**
**Mouse**	Mouse-*DUSP10*–F	CAGCCACTTCACATAGTCCTCG
	Mouse-*DUSP10*–R	GGGAGTTGTCACAGAGGTTTCC
	Mouse-*GLUT4*–F	GGTGTGGTCAATACGGTCTTCAC
	Mouse-*GLUT4*–R	AGCAGAGCCACGGTCATCAAGA
	Mouse-*microRNA-450a-5p*–F	UUUUGCGAUGUGUUCCUAAUAU
	Mouse-*U6*–F	CGCTTCGGCAGCACATATAC
	Mouse-*U6*–R	AAATATGGAACGCTTCACGA
	Mouse-*β-actin*–F	CATTGCTGACAGGATGCAGA
	Mouse-*β-actin*–R	CTGATCCACATCTGCTGGAA
**Human**	Human-*DUSP10*–F	ATCGGCTACGTCATCAACGTC
	Human-*DUSP10*–R	TCATCCGAGTGTGCTTCATCA
	Human-*GLUT4*–F	CCATCCTGATGACTGTGGCTCT
	Human-*GLUT4*–R	GCCACGATGAACCAAGGAATGG
	Human-*microRNA-450a-5p*–F	UUUUGCGAUGUGUUCCUAAUAU
	Human-*GAPDH*–F	TGTGGGCATCAATGGATTTGG
	Human-*GAPDH*–R	ACACCATGTATTCCGGGTCAAT

Supplementary Table 2 The antibodies of western-blot

Name	Catalog	manufacturer
JNK	9252S	Cell Signaling Technology
p-JNK	9255S	Cell Signaling Technology
IRS	2382S	Cell Signaling Technology
IRS-ser302	2384S	Cell Signaling Technology
IRS-ser307	2381S	Cell Signaling Technology
IRS-tyr895	3070S	Cell Signaling Technology
AKT	4685S	Cell Signaling Technology
AKT-ser473	4060S	Cell Signaling Technology
AKT-thr308	13038S	Cell Signaling Technology
GLUT4	ab33780	Abcam
DUSP10	ab71309	Abcam
PI3K	ab191606	Abcam
p-PI3K	ab182651	Abcam
GAPDH	TA-08	Zhongshan Golden Bridge Bio-technology
